# Current status of theranostics in prostate cancer

**DOI:** 10.1007/s00259-017-3882-2

**Published:** 2017-12-28

**Authors:** Irene Virgolini, Clemens Decristoforo, Alexander Haug, Stefano Fanti, Christian Uprimny

**Affiliations:** 1 Department of Nuclear Medicine, Medical University of Innsbruck, Anichstraße 35, 6020 Innsbruck, Austria; 2 Department of Radiology and Nuclear Medicine, Medical University of Vienna, 18–20 Währinger Gürtel, 1090 Vienna, Austria; 3 Nuclear Medicine Unit, University of Bologna, S. Orsola Hospital Bologna, Massarenti 9, 40138 Bologna, Italy

**Keywords:** ^68^Ga-PSMA, PET/CT, PET/MR, PET-guided personalized therapy, Theranostics, Prostanostics, Prostate cancer

## Abstract

The aim of this review is to report on the current status of prostate-specific membrane antigen (PSMA)-directed theranostics in prostate cancer (PC) patients. The value of ^68^Ga-PSMA-directed PET imaging as a diagnostic procedure for primary and recurrent PC as well as the role of evolving PSMA radioligand therapy (PRLT) in castration-resistant (CR)PC is assessed. The most eminent data from mostly retrospective studies currently available on theranostics of prostate cancer are discussed. The current knowledge on ^68^Ga-PSMA PET/CT implicates that primary staging with PET/CT is meaningful in patients with high-risk PC and that the combination with pelvic multi parametric (mp)MR (or PET/mpMR) reaches the highest impact on patient management. There may be a place for ^68^Ga-PSMA PET/CT in intermediate-risk PC patients as well, however, only a few data are available at the moment. In secondary staging for local recurrence, ^68^Ga-PSMA PET/mpMR is superior to PET/CT, whereas for distant recurrence, PET/CT has equivalent results and is faster and cheaper compared to PET/mpMR. ^68^Ga-PSMA PET/CT is superior to ^18^F / ^11^Choline PET/CT in primary staging as well as in secondary staging. In patients with biochemical relapse, PET/CT positivity is directly associated with prostate-specific antigen (PSA) increase and amounts to roughly 50% when PSA is raised to ≤0.5 ng/ml and to ≥90% above 1 ng/ml. Significant clinical results have so far been achieved with the subsequent use of radiolabeled PSMA ligands in the treatment of CRPC. Accumulated activities of 30 to 50 GBq of ^177^Lu-PSMA ligands seem to be clinically safe with biochemical response and PERCIST/RECIST response in around 75% of patients along with xerostomia in 5–10% of patients as the only notable side effect. On the basis of the current literature, we conclude that PSMA-directed theranostics do have a major clinical impact in diagnosis and therapy of PC patients. We recommend that ^68^Ga-PSMA PET/CT should be performed in primary staging together with pelvic mpMR in high-risk patients and in all patients for secondary staging, and that PSMA-directed therapy is a potent strategy in CRPC patients when other treatment options have failed. The combination of PSMA-directed therapy with existing therapy modalities (such as ^223^Ra-chloride or androgen deprivation therapy) has to be explored, and prospective clinical multicenter trials with theranostics are warranted.

## Introduction

Recognized imaging agents for prostate cancer (PC) include ^18^F- / ^11^C–choline, ^11^C–acetate, ^18^F–fluciclovine (FACBC), ^18^F-16β-fluoro-5α-dihydrotestosterone (FDHT), ^18^F–NaF and conventional ^99m^Tc-labeled phosphonates. The theranostic history of PSMA ligands started with radiolabeled anti-PSMA antibodies (e.g., Prostascint®) [1] first introduced more than 20 years ago at the John Hopkins’ University [2]. These compounds did not gain wide-spread clinical acceptance until 2012 when the first human studies with the ^68^Ga- PSMA-11-ligand [3] were performed at Heidelberg [4]. Within the last 5 years, the rapid development of different PSMA ligands and their clinical use has resulted in numerous publications, which established a new and comprehensive area of nuclear medicine from imaging to personalized peptide radionuclide ligand therapy (PRLT) of PC patients.

PSMA is a cell-surface enzyme that is continually internalized (synonym: glutamate carboxypeptidase II; folate hydrolase I; [5, 6]. This cell-surface protein (750 amino acids, 84 kDa) is overexpressed in PC [7] and its expression increases progressively in higher-grade tumors, metastatic or hormone-refractory disease, and under androgen deprivation therapy [ADT]. The level of PSMA expression is a significant indicator for disease outcome [8]. PSMA is not entirely prostate-specific, and it is expressed physiologically in normal cells including the small intestine, proximal renal tubulus, thyroid neoplasms, salivary and lacrimal glands (with potential impact on the side effect profile when used as targeting molecule) but also in other cancers such as renal cell cancer [9] due to an overexpression of PSMA on cancer-related neovascular structures.

A variety of PSMA ligands for PET as well as SPECT imaging have been introduced into the clinic over the recent years. Most literature exist for ^68^Ga-PSMA-11, but there are also some publications for ^68^Ga-PSMA-I&T and ^68^Ga-PSMA-617. The EANM and SNM guidelines [10] assume that the differences in the diagnostic capacity of these new radioligands are marginal, although no direct comparative studies are available.

In general, data from prospective multicenter trials are not yet available for ^68^Ga-PSMA ligands. None of these tracers has been approved, neither by the European Medicines Agency (EMA) nor the United States Food and Drug Administration (FDA). This is also a limitation within the most recent registration of the first ^68^Ge/^68^Ga generator describing “*a medicinal product which allows direct, simplified preparation of* ^*68*^ *Ga-radiopharmaceuticals in combination with licensed kits*” [11].

Since the life expectancy of patients with localized PC is more than 10 years [12], a careful choice of therapy approach is warranted. The National Comprehensive Cancer Network (NCCN) guidelines for PC [13] provide multidisciplinary recommendations on the clinical management of patients with PC based on clinical evidence and expert consensus. In newly diagnosed local PC, “active surveillance” and “watchful waiting” for low-risk patients is appropriate. Intermediate-risk patients have a risk of 3.7 to 20.1% for lymph node (LN) involvement and dissection should be performed if the risk exceeds 5% [14]. High-risk patients should undergo radical prostatectomy (RP) combined with extended LN dissection and locoregional radiation therapy (RT) [15]. Multimodality treatment is appropriate for high-risk disease including imaging procedures and long-term ADT. PC patients are generally treated by salvage RT to the prostate bed when local relapse is suspected and by ADT when systemic relapse is suspected. During follow-up, about 50% of patients treated initially by RP or RT experience biochemical recurrence (BR) [16]. Metastases-directed therapies could play a significant role in PC patients with CR if the imaging tool could accurately locate the lesions. ^11^C–Choline PET/CT has been proven to be a superior imaging tool compared to conventional imaging with significant impact on patient management despite of relatively low sensitivity in patients with low PSA levels [17].

Given that bone is the major site of distant metastases formation bone-targeted imaging and radionuclide therapy for bone pain palliation (^153^Sm- ethylendiamine-tetramethylen-phosphonate(EDTMP), ^223^Ra (Xofigo® [18]), ^177^Lu-labeled bisphosphonates [19], and direct bone marrow tumor cell killing [20]) have been implemented.

“Theranostic imaging” (therapy: *Greek therapeia:* to treat medically; knowledge: *Greek: gnosis*) refers to the combination of a predictive biomarker with a therapeutic agent [21]. The theranostic concept based on PSMA overexpression led to the use of PSMA ligands for systemic therapy in patients with castration-resistant (CR) PC. The aim of this review is to report on the current status of PSMA-directed theranostics in PC patients. The value of ^68^Ga-PSMA-directed PET imaging as a diagnostic procedure for primary and recurrent PC as well as the role of evolving peptide radioligand therapy (PRLT) in CRPC is assessed. The current available literature envisions that the theranostics of PC will be commonplace in the personalized care of men.

## Radiopharmaceuticals

Table 1 gives an overview of PSMA ligands that have been studied in PC patients both for diagnosis and therapy. A variety of other PSMA-targeting radiopharmaceuticals have been reported, but so far not been evaluated in patients.

**Table 1:** PSMA ligands used in patients - status October 2017

COMPOUND	RADIONUCLIDE	REFERENCE
*“DUPA”-based*		
PSMA-11 (PSMA HBED-CC)	^68^Ga	Eder et al. [3] Afshar-Oromieh et al. [4]
PSMA-617	^177^Lu, ^225^Ac, ^64^Cu (^68^Ga, ^111^In)	Afshar-Oromieh et al. [22] Benesova et al. [23]
PSMA-I&T	^68^Ga, ^177^Lu, ^111^In	Weineisen et al. [24]
PSMA-I&S	^99m^Tc	Robu et al. [25]
MIP-1404/1405/1427	^99m^Tc	Hillier et al. [26]
MIP-1095	^131^I (^124^I)	Barret et al. [27]
DCFBC	^18^F	Cho et al. [28]
DCFPyL	^18^F	Chen et al. [29]
PSMA-1007	^18^F	Cardinale et al. [30] Giesel et al. [31]
*Antigen-targeted*		
Capromab pendetide (ProstaScint®)	^111^In	Manyak [32]
J591	^111^In, ^90^Y, ^177^Lu, ^89^Zr	Bander et al. [2]
IAB2M	^89^Zr	Pandit-Taskar et al. [33]

The current clinical success of radiolabeled PSMA ligands is based on a small motif binding to the catalytic *N*-acetyl-l-aspartyl-l-glutamate hydrolyzing site in the PSMA molecule. This 2-[3-(1,3-dicarboxypropyl)ureido]pentanedioic acid (DUPA) motif was first described by Kozikowski et al. [34]. Eder et al. [3] introduced a specific chelator for gallium, *N*,*N*′-Bis(2-hydroxy-5-(ethylene-beta-carboxy)benzyl)ethylenediamine *N*,*N*′-diacetic acid(HBED-CC) via a Lys-Ahx linker resulting in the compound PSMA-11, and showed that the lipophilicity of the HBED-CC-chelator revealed superiority over well-established 1,4,7,10-tetraazacyclododecane-1,4,7,10-tetraacetic acid (DOTA), at the same time maintaining high-affinity to PSMA [22]. As HBED-CC only binds ^68^Ga and not other trivalent radiometals such as ^177^Lu or ^111^In, the same authors developed a specific DOTA-based compound, PSMA-617 [23] by introducing a p-iodo phenyl substitution in the linker between the DUPA motif and DOTA ensuring the required lipophilicity in the side chain. Early patient studies with ^177^Lu-PSMA-617 [35] confirmed the high uptake by PSMA-expressing tumors and at the same time showing reduced kidney retention as compared to PSMA-11, which makes this ligand suitable for radionuclide therapy applications. However, as the reduced kidney retention is based on slower pharmacokinetics, the imaging properties seem to be inferior compared to ^68^Ga-PSMA-11. PSMA-617 was also applied in patients labeled with ^225^Ac for therapy [36] and ^64^Cu for diagnosis [37]. In parallel, Weineisen et al. [24] reported on another DOTA-PSMA ligand, “PSMA-I&T”, with two phenyl substitutions in the side chain, but also based on the DUPA motif. This compound was labeled both with ^68^Ga and ^177^Lu for theranostic applications and was shown to efficiently target PSMA-expressing tumors in PC patients. Recently, the same group has reported PSMA-I&S to be labeled with ^99m^Tc [25]. It is based on the DUPA motif with a mercaptoacetyltriserine (MAS3) as chelating moiety for ^99m^Tc involving a linker with a lipophilic naphthyl and Tyr residue. Already Molecular Insight Pharmaceuticals developed a series of ^99m^Tc-PSMA ligands based on the DUPA motif and introduced it into prospective clinical trials [26], two compounds, MIP-1404 and MIP-1405, both based on an imidazole modification for binding the Tc-tricarbonyl-core. The same company also developed radioiodinated compounds using a *p*-iodo phenyl substitution. One of them, MIP-1095, showed excellent targeting properties when labeled with ^123^I [27] and was used subsequently labeled with ^131^I for therapy studies.

Furthermore, ^18^F–labeled compounds have been developed and used for PSMA imaging. The first were developed by the group of Pomper et al. [28] at the John Hopkins University, using a F-fluorobenzyl-l-cysteine attachment to the DUPA motif, called ^18^F–DCFBC. A further development, ^18^F–DCFpyl with a fluoro-nicotinic acid substitution [29], was introduced to clinical trial by Progenics Pharmaceuticals. Most recently, the group in Heidelberg presented an ^18^F–labeled version, PSMA-1007, introducing a lipophilic spacer with naphthyl substitution [30] and showing comparable imaging performance in patients to ^68^Ga-PSMA-11 [31].

Before the DUPA motif was described to target PSMA in a highly specific way, already other strategies have been pursued in the attempt to develop PSMA-targeting radiopharmaceuticals. In particular, antibody-based constructs were developed for radiolabeling directed against the PSMA-protein. ^111^In-capromab pendetide (ProstaScint®), based on the murine monoclonal antibody 7E11-C5.3, was widely used, particularly in the US for SPECT imaging of PC [32]. It is directed against an intracellular domain of PSMA, therefore not reaching high sensitivity in imaging. This concept was further developed towards the humanized antibody J591, directed against the extracellular domain of PSMA and was labeled with ^111^In for dosimetry and ^90^Y as well as ^177^Lu for therapeutic applications [2]. It showed promising results in a number of clinical trials. As intact antibodies exhibit well-known limitations regarding slow tumor targeting and delayed clearance from non-target tissue, recently Pandit-Tasker et al. [33] reported on the application of the minibody IAB2M derivatized with desferrioxamine for ^89^Zr labeling. This 80-kDa molecule, genetically engineered from the intact antibody J591 (150 kDa), lacking the Fc-receptor interaction domains and making it pharmacologically inert, showed favorable biodistribution and kinetics for targeting metastatic PC in this phase I trial [32]. Imaging at 48 h p.i. provided good lesion visualization when labeled with ^89^Zr for PET.

## Primary PC - “primary imaging”

According to the European Association of Urology (EAU)-European Society for Radiotherapy & Oncology (ESTRO)-International Society of Geriatric Oncology (SIOG) guidelines [15], in high-risk localized PC or high-risk locally advanced PC, staging should be performed with pelvic mpMRI and cross-sectional abdominal pelvic imaging and bone scanning for metastatic screening. These imaging procedures may eventually also be useful in patients with intermediate-risk PC, whereas the guidelines do not recommend additional imaging for staging purpose in low-risk PC patients as the accuracy of conventional imaging procedures is limited especially regarding the detection of small LN.

In primary PC, the diagnostic accuracy of ^68^Ga-PSMA-ligand PET/CT is not yet proven and only a few studies have been published so far. Along with the first registered ^68^Ge/^68^Ga generator [11] by Galliapharm, a prospective European multicenter trial is finally under way in high-risk PC patients with a Gleason Score (GS) > 7 [38].

Sachpekidis et al. [39] aimed to retrospectively assess the pharmacokinetics and biodistribution of Ga-PSMA-11 in 24 patients suffering from primary PC by means of dynamic (pelvic) and whole-body PET/CT. Overall, 23/24 patients (95.8%) were ^68^Ga-PSMA-11 PET positive and in 9/24 patients (37.5%) metastatic lesions were detected. Time–activity curves derived from PC-associated lesions revealed an increasing Ga-PSMA-11 accumulation during the dynamic PET acquisition procedure.

In line with these observations, we retrospectively investigated the value of ^68^Ga-PSMA-11 PET/CT in primary staging of PC [40] in 90 patients (GS 6–10; median prostate-specific antigen (PSA): 9.7 ng/ml) with transrectal ultrasound (TRUS)-guided biopsy-proven PC. The SUV_max_ of the primary tumor was assessed in relation to both PSA level and GS. Eighty-two patients (91.1%) demonstrated pathologic tracer accumulation in the primary tumor that exceeded the physiologic tracer uptake in normal prostate tissue (median SUV_max_: 12.5 vs. 3.9). Tumors with GS of 6, 7a (3 + 4) and 7b (4 + 3) showed significantly lower ^68^Ga-PSMA-11 uptake, with median SUV_max_ of 5.9, 8.3, and 8.2, respectively, compared to patients with GS > 7 (median SUV_max_: 21.2; *p* < 0.001). PC patients with PSA ≥10.0 ng/ml exhibited significantly higher uptake than those with PSA-levels < 10.0 ng/ml (median SUV_max_: 17.6 vs. 7.7; *p* < 0.001). In 24/90 patients (26.7%), 82 LN with pathologic tracer accumulation consistent with metastases were detected (median SUV_max_: 10.6). Eleven patients (12.2%) revealed 55 pathologic bone lesions suspicious for bone metastases (median SUV_max_: 11.6). The results allow the conclusion that ^68^Ga-PSMA-11 PET/CT should be preferentially applied for primary staging in patients with GS > 7 or PSA-levels ≥10 ng/ml.

Maurer et al. [41] retrospectively evaluated the diagnostic efficacy of ^68^Ga-PSMA-11 PET compared to conventional imaging (CT/mpMRI) for LN-staging in 130 consecutive patients with intermediate- to high-risk PC prior to RP. LN metastases were found in 41/130 patients (31.5%). On patient-based analysis the sensitivity, specificity, and accuracy of ^68^Ga-PSMA-11 PET were 65.9, 98.9, and 88.5%, and those of morphological imaging were 43.9, 85.4, and 72.3%, respectively. Of 734 dissected LN templates, 117 (15.9%) showed metastases. On template-based analysis, the sensitivity, specificity, and accuracy of ^68^Ga-PSMA-11-PET were 68.3, 99.1, and 95.2%, and those of morphological imaging were 27.3, 97.1, and 87.6%, respectively. The results demonstrate that in patients with intermediate- to high-risk PC, preoperative LN staging with ^68^Ga-PSMA-11-PET is superior to standard routine imaging and thus has the potential to replace current standard imaging for this indication.

Budäus et al. [42] retrospectively compared preoperative ^68^Ga-PSMA PET/CT LN findings with histologic work-up after RP in 30 patients and found that LN metastasis detection rates were substantially influenced by LN metastases size. In 92.9% of patients, the intraprostatic tumor foci were correctly predicted. Overall, 608 LNs containing 53 LN metastases were detected. LN metastases were present in 12/30 patients (40%), which were found by ^68^Ga-PSMA PET/CT in four patients (33.3%). Median size of ^68^Ga-PSMA-PET/CT-detected vs. undetected LN metastases was 1.36 vs. 0.43 cm (*p* < 0.05). Overall sensitivity, specificity, positive predictive value, and negative predictive value of ^68^Ga-PSMA PET/CT for LN metastases detection were 33.3, 100, 100, and 69.2%, respectively. Per-side analyses revealed corresponding values of 27.3, 100, 100, and 52.9%. Compared to Maurer et al. [41], who reported a higher sensitivity, the limitations of this retrospective assessment are not only the smaller number of pooled patients from five different institutions but also differences in the report protocols, which may lead to variations in the assessment and thus lower sensitivity [43]. On the other hand, also van Leeuwen et al. [44], who performed a prospective study in 30 intermediate- to high-risk patients, reported size-dependence of positively imaged LN.

In a cohort of 34 PC patients, Herlemann et al. [45] reported a sensitivity of 84%, specificity of 82%, PPV of 84%, and NPV of 82% for detection of LN in patients with intermediate- to high-risk PC. Postoperative histopathology was taken as a reference standard after primary (*n* = 20) or secondary LN dissection (*n* = 14). ^68^Ga-PSMA-11 PET/CT detection rates were superior to CT alone before primary (sensitivity 88 vs. 75%) as well as secondary (sensitivity 77 vs. 65%) LN dissection in 14 patients.

Upon initial staging, Demirkol et al. [46] in eight patients, Sterzing et al. [47] in 15 patients, and Sahlmann et al. [48] in 12 patients confirmed the potential value of ^68^Ga-PSMA-11 PET.

## Recurrent PC - “secondary staging”

In PC with BR after primary therapy, conventional imaging techniques have a low detection rate at the PSA levels at which targeted therapy with curative intent, such as salvage radiotherapy is effective. A magnitude of data indicate that ^68^Ga-PSMA-PET can detect recurrent PC or small LN metastases that are ^18^F–choline PET-negative.

Recently, Perera et al. [49] overviewed mostly retrospective data from 16 studies on ^68^Ga-PSMA PET efficiency in PC patients with rising PSA values. At BR, a pooled PET-detection rate of 76% was reported for PSA ranges of 1–2 ng/ml and of 58% for PSA ranges of 0.2–1.2 ng/ml, which demonstrates the improved diagnostic performance for PC patients. Most literature exists for ^68^Ga-PSMA-11 compared with ^68^Ga-PSMA-I&T and ^68^Ga-PSMA-617. These data are somewhat critical, as the authors summarize results collected from studies using different peptides, i.e., PSMA-11, PSMA-I&T, and PSMA-617, and furthermore from mixed patient populations as well. The authors also did not take into account important features of imaging protocols such as imaging time or medication (i.e., ADT).

The first data by Ceci et al. [50] in 70 consecutive PC patients identified an association of PSA level and PSA kinetics in terms of PSA_doubling time (dt)_ with a pathological ^68^Ga-PSMA-11 PET/CT in PC patients with BR after RP. A positive PET scan was observed in the PSA range 0.14 to 35.07 ng/ml (median, 2.39) and a negative PET scan in the range 0.21 to 5.00 ng/ml (median, 0.81 ng/ml). ROC analysis showed that a PSA_dt_ of 6.5 months and a PSA of 0.83 ng/ml were optimal cut-off values for ^68^Ga-PSA PET-positivity, which was observed in 17 of 20 patients (85%) with PSA < 2 ng/ml and PSA_dt_ > 6.5 months.

Verburg et al. [51] retrospectively investigated 155 patients. PET/CT was positive in 44, 79, and 89% of patients with PSA levels of ≤1, 1–2, and ≥2 ng/ml, respectively. Patients with high PSA levels showed higher rates of local PC tumors (*p* < 0.001), extrapelvic LN (*p* = 0.037), and bone metastases (*p* = 0.013). A shorter PSA_dt_ was significantly associated with pelvic LN (*p* = 0.026), extrapelvic LN (*p* = 0.001), bone (*p* < 0.001), and visceral (*p* = 0.041) metastases. A high GS was associated with more frequent pelvic LN metastases (*p* = 0.039). In multivariate analysis, both PSA and PSA_dt_ were independent determinants of scan positivity and of extrapelvic LN metastases. These data show that higher PSA levels and shorter PSA_dt_ are independently associated with scan positivity and extrapelvic metastases, and can be used for patient selection for ^68^Ga-PSMA-11 PET.

^68^Ga-PSMA-11 PET/CT-guided salvage retroperitoneal LN dissection for disease relapse after RP was first reported in 2015 [52]. ^68^Ga-PSMA has a high detection rate of PC recurrence outside the prostatic fossa in patients being considered for salvage RT. In fact, ^68^Ga-PSMA-11 PET/CT appears to be useful for re-staging of PC in patients with rising PSA who are being considered for RT even at PSA levels < 0.5 ng/ml. The only available prospective study by van Leeuwen et al. [53] in a total of 300 consecutive patients considered for salvage RT identified 70 patients with a BR of PSA ≥0.05 and < 1.0 ng/ml after RP. Among patients with PSA levels of 0.05 to 0.09 ng/ml, 8% were definitely positive; the corresponding percentages for the other PSA ranges were as follows: PSA 0.1 to 0.19 ng/ml, 23%; PSA 0.2 to 0.29 ng/ml, 58%; PSA 0.3 to 0.49 ng/ml, 36% and PSA 0.5 to 0.99 ng/ml, 57%. Noteworthy, as a result of the ^68^Ga-PSMA PET-findings, the authors report a major management change in 20 (28.6%) patients, which will have future importance of changes in the RT volume to be applied.

Afshar-Oromieh et al. retrospectively [54] investigated 319 patients of whom 82.8% had at least one ^68^Ga-PSMA-11 PET-positive lesion. Tumor detection was positively associated with PSA level and ADT, whereas GS and PSA_dt_ were not associated with tumor detection. Among lesions investigated by histology, 30 were false-negative in four different patients, and all other lesions (*n* = 416) were true-positive or true-negative. A lesion-based analysis of sensitivity, specificity, negative predictive value (NPV), and positive predictive value (PPV) revealed values of 76.6, 100, 91.4, and 100%. A patient-based analysis revealed a sensitivity of 88.1%. Of 116 patients available for follow-up, 50 received local therapy after ^68^Ga-PSMA PET/CT. In the range of PSA < 0.2 ng/ml, the scan was positive in 8/17 patients (47.1%). The authors concluded that PET/CT can help to delay systemic therapy of PC. In their recent report, Afshar-Oromieh et al. [55] retrospectively analyzed 1007 patients with BR. In 801/1007 (79.5%) of patients, at least one lesion was detected on ^68^Ga-PSMA-11 PET/CT and scan sensitivity was significantly associated with PSA level and ADT. Multivariate analysis found, however, no relevant correlation with PSA_dt_ or PSA velocity as well as GS and PET positivity. GS and amount of injected activity were not associated with PET positivity. Noteworthy, in patients with PSA < 0.2 ng/ml 32/69 (46%), PET-scans were positive and 15 patients had PSA levels < 0.1 ng/ml.

Eiber et al. [56] investigated 248 patients with PC after RP and found pathological findings in 202/248 (89.5%) by ^68^Ga-PSMA-11 PET/CT. The detection rates were 96.8, 93.0, 72.7, and 57.9% for PSA levels of ≥2, 1 to < 2, 0.5 to < 1, and 0.2 to < 0.5 ng/ml, respectively. Whereas detection rates increased with a higher PSA velocity, no significant association could be found for PSA_dt_. ^68^Ga-PSMA-ligand PET (as compared with CT) exclusively provided pathologic findings in 81 (32.7%) patients. In 61 (24.6%) patients, it exclusively identified additional involved regions. In patients with higher GS (≤7 vs. ≥8), detection efficacy was significantly increased (*p* = 0.0190), however, not with ADT. In a recent report [57], the same group, however, reported in a more homogeneous cohort of patients after RT a significant higher detection rate for patients under ADT (*p* = 0.0381; 44/45 [97.7%] vs. 63/73 [86.3%]) and positive association with increasing PSA levels. The detection rates were 81.8 (36/44), 95.3 (41/43), and 96.8% (30/31) for PSA of 2 to < 5, 5 to < 10, and ≥10 ng/ml, respectively (*p* = 0.0377). ^68^Ga-PSMA ligand PET/CT indicated local recurrence in 68 of 107 patients (63.5%), distant lesions in 64 of 107 patients (59.8%), and local recurrence as well as distant lesions in 25 of 107 patients (23.4%).

Kabasakal et al. [58] reported a PET positivity of 31% (*n* = 4), 54% (*n* = 13), and 88% (*n* = 14) in patients with a PSA level of less than 0.2, 0.2–2, and 2–5 ng/ml, respectively. A positive correlation was also observed between positivity and GS. According to patient-based analysis, a sensitivity of 76.5% and a specificity of 91.7% were found.

Sachpekidis et al. [59] found in 22/31 (71.0%) patients with BR after RP a ^68^Ga-PSMA-11-positive scan. The median PSA value in the ^68^Ga-PSMA-11-positive group was significantly higher (median = 2.35 ng/ml; range = 0.19–130.0 ng/ml) than in the ^68^Ga-PSMA-11-negative group (median value: 0.34 ng/ml; range, = 0.10–4.20 ng/ml). Time–activity curves derived from PC recurrence-indicative lesions revealed an increasing ^68^Ga-PSMA-11 accumulation during dynamic PET acquisition over 60 min.

The limited value of conventional CT and MR in the detection of local recurrence and LN metastases is well known [60]. In 48 patients with BR and a median PSA of 1.31 ng/ml, Rauscher et al. [61] compared ^68^Ga-PSMA PET to CT or MRI and histopathology following salvage lymphadenectomy. PET detected 53/68 histologically proven LN fields (78%), whereas morphological imaging was positive in only 18/67 (27%) resulting in a *p* < 0.001. PET-positive LN had a mean size of 8.3 ± 4.3 mm (range, 4–25 mm).

Albisinni et al. [62] recently reported subsequent change in management in 99/131 (76%) of patients imaged after RP, RT, or both, for BR. The authors found a positive scan in 45% of patients with a PSA level of ≤0.5 ng/ml and in 75% with PSA level of 0.5 to 1.0 ng/ml.

## Performance of ^68^Ga-PSMA PET/CT versus PET/MR 

Lesion detectability increases with acquisition time, reaching its maximum at PET acquisition time of 4 min per PET position [63]. Furthermore, PET-acquisition duration has a significant impact on the incidence of the halo artifact around kidneys and bladder, decreased lesion detectability and lower SUV, as well as lower arm attenuation values [64]. Positioning the arms down was shown to be significantly associated with the appearance of the halo artifact [65].

### Primary staging

**Fig. 1:**
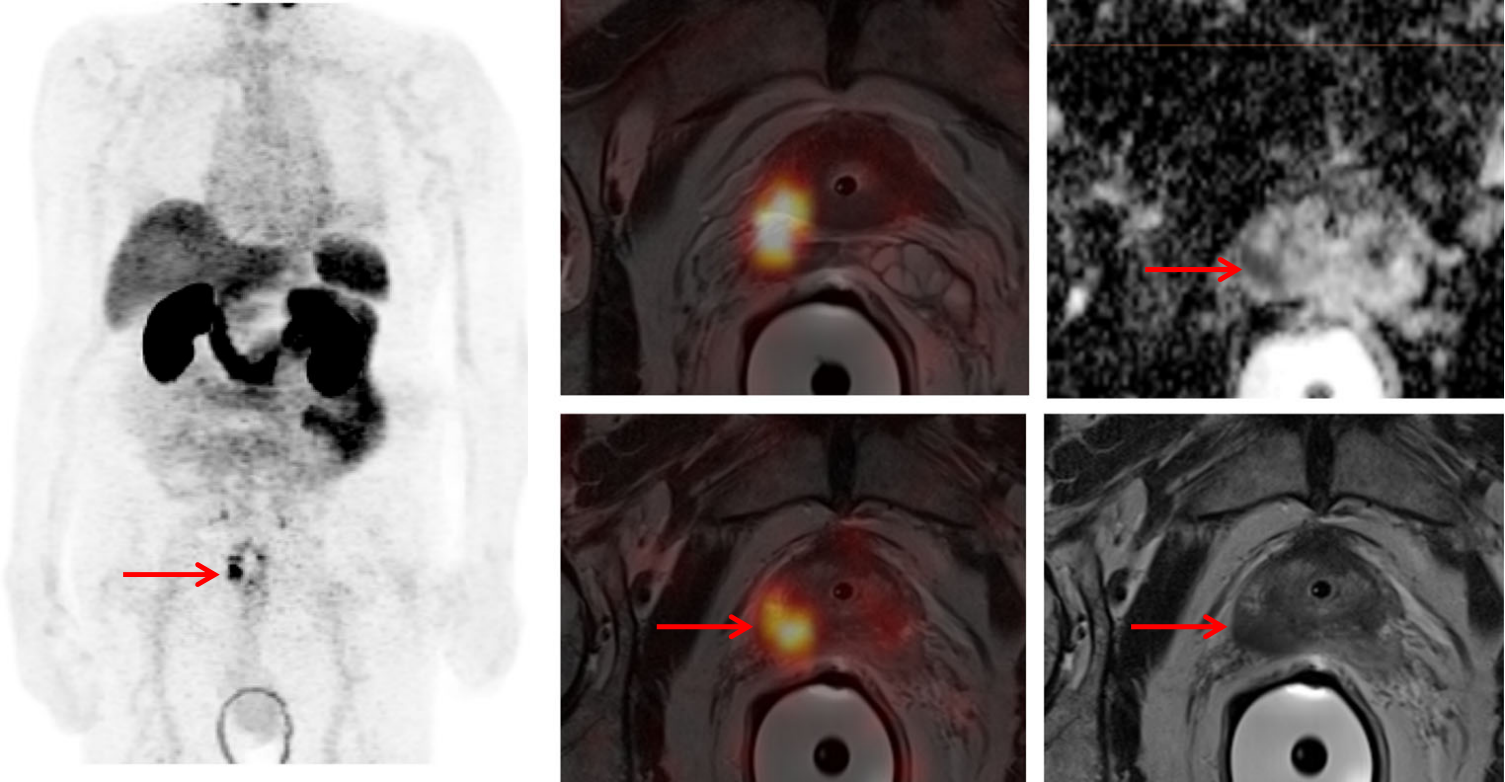
PET/MRI demonstrating the primary PC (PSA 16 ng/ml, GS 3 + 4) in the right prostate lobe (*red arrow*) invading the seminal glands with markedly increased ^68^Ga-PSMA uptake. The tumor presents with restricted diffusion on apparent diffusion coefficient (ADC) mapping and is hypointense on T2-weighted MRI

Simultaneous ^68^Ga-PSMA PET/mpMRI may improve the localization of primary PC (Fig. 1). Eiber et al. [66] investigated 53 patients in whom 202 of 318 sextants (63.5%) contained PC at pathologic examination following RP. Simultaneous PET/mpMRI statistically outperformed mpMRI (*p* < 0.001) and PET imaging (*p* = 0.002) for localization of PC. Compared with mpMRI, PET imaging was more accurate (*p* = 0.003) and provided a high uptake ratio between malignant vs. non-malignant tissue (i.e., 5.02 [range, 0.89–29.8]), but no significant correlation was observed between quantitative PET parameters and GS or PSA value.

In 92 consecutive patients with intermediate- to high-risk PC, Maurer et al. [67] reported for 470 anatomical fields with 52 LN metastases a sensitivity of 73.1%, specificity of 98.6%, accuracy of 57.7%, PPV of 86.4%, and NPV of 96.7%, surpassing reported results for standard imaging.

Giesel et al. [68] showed that ^68^PSMA PET/CT and mpMRI correlated well with regard to tumor allocation in patients with high pretest probability for large primary tumors upon initial staging. A combination of both methods performed even better in terms of sensitivity and specificity as demonstrated by Zamboglou et al. [69, 70] and may thus have a potential role in RT planning. For 89.4% of sections containing a tumor according to mpMRI the tumor was also identified in total or near-total agreement by PSMA PET. Vice versa, for 96.8% of the sections identified as tumor bearing by PSMA PET, the tumor was also found in total or near-total agreement by mpMRI.

An ongoing study sponsored by Stanford University [71] in patients with intermediate- and high-risk PC is currently evaluating the clinical usefulness of ^68^Ga-PET/mpMRI with an estimated date 2021 for final data collection for primary outcome measure.

### Secondary staging

Kranzbühler et al. [72] suggested an improved allocation of PSMA activity with soft tissue versus urine in the pelvic area using PET/mpMRI. Overall, in 44/56 patients (79%), PET/mpMR was positive - in 4/9 patients (44%) with PSA values of 0.05 to 0.2 ng/ml and in 9/12 patients with PSA values of 0.2 to 0.5 ng/ml, which is significantly higher as reported for PET/CT, which was positive in 57.9% [54] in the range of 0.2 to 0.5 ng/ml (*p* = 0.001).

The proportion of discordant PSMA-positive suspicious findings in PET/CT versus PET/mpMRI was very low as investigated by Freitag et al. [73]. In their study, 98.5% of 64 LNs and 100% of 28 skeletal lesions were concordant. Furthermore, in 18/119 patients (15.1%) PET/mpMRI identified local recurrence whereas PET/CT was positive in nine patients only [74].

Especially, in patients with low PSA values, the diagnostic certainty was substantially higher in PET/MR (*n* = 76) compared to PET/CT (*n* = 256) as demonstrated by Maurer et al. [75] in a total of 332 patients: for PSA values 0.2–0.5 ng/ml 38.5 vs. 69.2% of positive findings on PET/CT vs. PET/MR were rated as highly suggestive for PC recurrence.

## ^68^Ga-PSMA versus ^11^C- / ^18^F–flouromethyl-choline PET/CT

Despite the fact that the guidelines of the EAU [15] as well as the National Comprehensive Cancer Network (NCCN) [13] suggest the use of ^18^F- or ^11^C–choline PET in PC patients with recurrent disease, the accuracy of choline PET is not well assessed, as most published studies are retrospective. The detection rate for ^11^C–choline-PET/CT in patients with PSA < 1.0 ng/ml was 19% in 51 patients [76]. In the largest study published so far [77], reporting more than 4000 ^11^C–choline PET/CT scans, the detection rate was 27% in patients with PSA < 1.16 ng/ml.

Fanti et al. [78] found in a comprehensive literature search 425 studies and finally analyzed 18 articles critically, which evaluated the role of ^11^C–choline PET/CT at initial staging of PC in a total of 2126 patients providing a pooled detection rate of 62%. In 12 articles with 1270 participants, the pooled sensitivity was 89% and the pooled specificity was 89%. For LN disease in seven studies with 752 participants, the pooled detection rate was 36%.

A similar meta-analysis was published by Evangelista et al. [79] for intermediate- to high-risk PC patients using either ^18^F- or ^11^C–choline PET/CT. The meta-analysis included ten selected studies with a total of 441 patients and showed a pooled sensitivity of 49% and a pooled specificity of 95%.

**Fig. 2:**
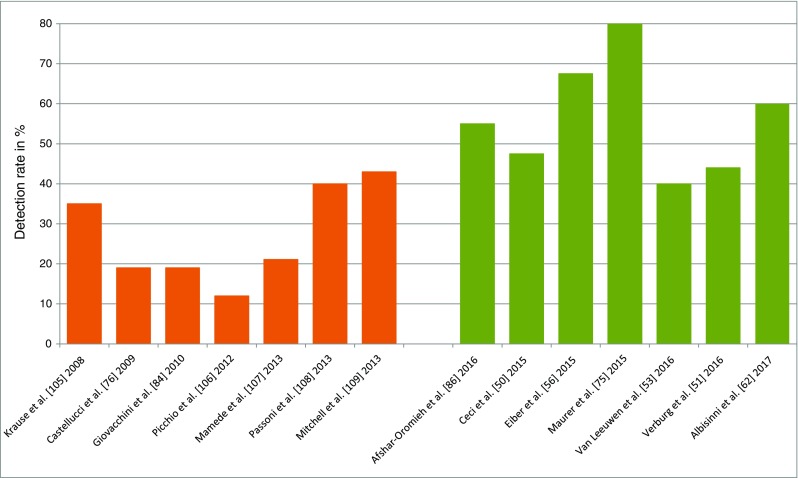
Detection rate in PC patients with low biochemical relapse of PSA < 1.0 ng/ml scanned either by ^18^F/^11^C–choline (*orange bars*) or ^68^Ga-PSMA-ligand (*green bars*)

^68^Ga-PSMA PET/CT was superior to ^18^F–choline in patients with BR and identified in 43.8% of patients recurrent disease, which was ^18^F–choline -negative [80]. Similar data stating a better detection rate of ^68^Ga-PSMA PET/CT as compared to choline PET/CT have been reported by other authors [81, 82]. To indirectly compare PSMA and choline as PET/CT tracers to identify lesions in patients with early BR, we reviewed the published data stratified by PSA values (Fig. 2). As demonstrated, the performance of PSMA PET/CT is superior to choline PET/CT, with an estimated detection rate of 40–60% at early BR (PSA < 1.0 ng/ml), and this assumption is also confirmed in a recent meta-analysis [83].

A recent reappraisal of 20 years of clinical PET/CT studies with choline presented by Giovacchini et al. [84] summarizes that choline-PET/CT should be used in BR patients with PSA > 1 ng/ml, pointing to the “undoubted” fact that ^68^Ga-PSMA PET/CT may be more promising for centers with the required technical equipment. The only available prospective study that compared ^68^Ga-PSMA-11 with ^18^F–choline PET/CT in PC patients with rising PSA after curative treatment was reported by Morigi et al. [85]. They imaged 38 patients, 34 (89%) had undergone RP and four (11%) had undergone RT. The scan results were positive in 26 patients (68%) and negative with both tracers in 12 patients (32%). Of the 26 positive scans, 14 (54%) were positive with ^68^Ga-PSMA alone, 11 (42%) with both ^18^F–choline and ^68^Ga-PSMA, and only one (4%) with ^18^F–choline alone. When PSA was below 0.5 ng/ml, the detection rate was 50% for ^68^Ga-PSMA versus 12.5% for ^18^F–choline. When PSA was 0.5–2.0 ng/ml, the detection rate was 69% for ^68^Ga-PSMA versus 31% for ^18^F–choline, and when PSA was above 2.0, the detection rate was 86% for ^68^Ga-PSMA versus 57% for ^18^F–choline. On lesion-based analysis, ^68^Ga-PSMA detected more lesions than ^18^F–choline (59 vs. 29, *P* < 0.001). There was a 63% (24/38 patients) management impact, with 54% (13/24 patients) being due to ^68^Ga-PSMA imaging alone.

Pfister et al. [80] compared ^68^Ga-PSMA-11 PET results in 38 patients with ^18^F–choline PET results in 28 patients before salvage lymphadenectomy using histology as the standard. For ^18^F–choline and ^68^Ga-PSMA-11, the respective sensitivity was 71.2 and 86.9%, specificity was 86.9 and 93.1%, PPV was 67.3 and 75.7%, NPV was 88.8 and 96.6%, and accuracy was 82.5 and 91.9% identifying a better performance of ^68^Ga-PSMA-11 PET/CT for the detection of locoregional recurrent and/or metastatic lesions prior to salvage lymphadenectomy.

## Factors associated with sensitivity of imaging

### Imaging protocol (early/dynamic, standard and delayed imaging)

After injection of ^68^Ga-PSMA-11, the “uptake time” is 1 h with an acceptable range of 50 to 100 min [34] although no standard imaging protocol has yet been defined. The intense physiological urinary bladder activity at 1 h post-injection presents a problem in the assessment of local relapse (prostate bed and vicinity).

The current data suggest that early dynamic imaging improves the detection rate of local recurrence and thus should be performed in addition to whole-body imaging at 1-h post-injection in PC patients with BR. Delayed imaging may be helpful as well, dependent on the administered activity and difficult to perform in the routine clinical setting as well.

A clinical impact of additional late imaging at 3 h post-injection was reported by Afshar-Oromieh et al. [55, 86] for most PC lesions as they show an increased uptake and a better lesion-to-background contrast compared to PET images acquired at 60 min p.i. Increased uptake of histologically confirmed tumor lesions with overtime was also found by Sahlmann et al. [48] in patients with recurrent PC and high-risk PC. In addition, tracer accumulation within the urinary bladder is lower at 3 h p.i., especially when furosemide is applied. Consequently, scans acquired at 3 h p.i. detect more tumor lesions than at 1 h. Using ^68^Ga-PSMA-I&T, Schmuck et al. [87] compared standard and delayed imaging in patients with BR or PSA persistence after primary therapy of PC. They found that delayed imaging can detect PC lesions with increased uptake compared to standard imaging in a small proportion of patients with 10/184 (5.4%) positive ^68^Ga-PSMA-I&T scans exclusively only at 3-h post-injection (*p* = 0.35).

**Fig. 3:**
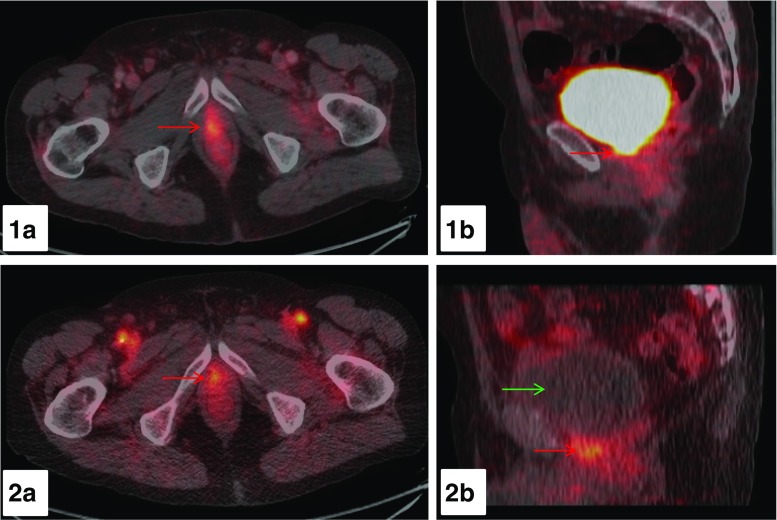
^68^Ga-PSMA-11 PET/CT images of a 72-year-old PC-patient with BR after RP (PSA 4.26 ng/ml). Early dynamic imaging of the pelvis over the first 8 min p.i. and a whole-body scan at 60-min p.i. were performed. At 60-min p.i., a clear distinction between urinary activity within the neck of the urinary bladder and local recurrence is not possible as presented on axial (1a) and sagittal (1b) fused PET/CT images (*red arrow*). In contrast, on the axial and sagittal fused PET/CT-images (2a, 2b) at 4 min p.i. of the early dynamic PET-acquisition a focal tracer accumulation with a SUV_max_ value of 4.45 adjacent to the urinary bladder is visible (*red arrow*) with no tracer uptake in the urinary bladder present (*green arrow*) consistent with local recurrence

With respect to assessment of local recurrence, we found in a retrospective analysis of 80 PC-patients that early dynamic imaging starting immediately after injection of ^68^Ga- PSMA-11 PET/CT allows the discrimination of urinary bladder activity from PC lesions [88]. A total of 55 lesions consistent with malignancy on 60-min whole-body imaging exhibited also pathologic ^68^Ga-PSMA-11 uptake during early dynamic imaging of the pelvic area (prostatic bed/prostate gland: *n* = 27; LN: *n* = 12; bone: *n* = 16). All pathologic lesions showed tracer uptake within the first 3 min, whereas urinary bladder activity was absent within the first 3 min of dynamic imaging in all patients. SUV_max_ was significantly higher in PC lesions within the first 6 min compared to urinary bladder accumulation (*p* < 0.001, [Fig. 3]). An early onset of tracer accumulation at typical sites of local recurrence before tracer activity is visible in the urinary bladder is characteristic for LR. Applying these criteria in the subgroup of PC patients with BR the detection rate of local recurrence was substantially higher with early dynamic imaging compared to PET scans 60-min p.i. (29.7 vs. 20.3%). These results are in line with the observation of Kabasakal et al. [89] who also concluded that early imaging could be helpful in the assessment of the prostate bed and structures in the proximity of the urinary bladder.

### Image interpretation

A recent multicenter study [43] retrospectively standardized image-interpretation criteria for ^68^Ga-PSMA PET/CT to detect recurrent PC in patients treated with primary curative intent (RP or RT) who presented a BR. On the basis of the consensus readings, criteria for ^68^Ga-PSMA PET/CT interpretation were defined. Between-reader agreement for the presence of anomalous findings in any of the five sites was only moderate. The agreement improved and became substantial when readers had to judge whether the anomalous findings were suggestive for a pathologic, uncertain, or non-pathologic image, and after a second Delphi round only four cases of disagreement remained. By developing these consensus guidelines on the interpretation of ^68^Ga-PSMA PET/CT, clinicians reporting these studies will be able to provide more consistent clinical reports and that within clinical trials, abnormality classifications will be harmonized, allowing more robust assessment of its diagnostic performance.

Interobserver agreement for ^68^Ga-PSMA-11 PET/CT study interpretations was recently also evaluated by Fendler et al. [90] who showed a highly consistent interpretation among observers with high levels of experience, suggesting that initial training on at least 30 patient cases is recommended to ensure acceptable performance.

### Influence of ADT on SUV_max_ and lesion detection rate

**Fig. 4:**
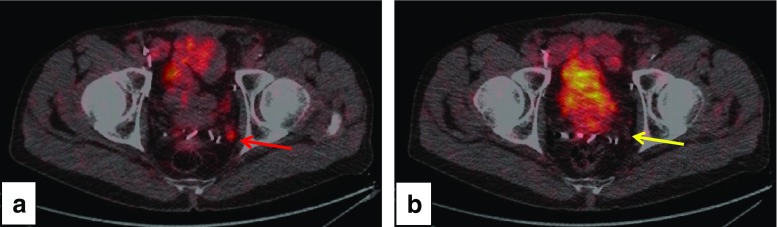
Potential effect of bicalutamide on ^68^Ga-PSMA-11 PET-uptake. On initial PET/CT (**a**, *red arrow*) moderately increased tracer accumulation with a SUV_max_ of 4.47 in a LN (5.7 mm in diameter) was found in the region of the left internal iliac vessels as shown on axial fused PET/CT-images. On follow-up PET/CT performed 7 days after initiation of bicalutamide (160 mg/day; 1 week), no pathologic tracer accumulation was found in the left iliac LN, which morphologically remained unchanged (**b**; *yellow arrow*). PSA decreased from 0.94 to 0.18 ng/ml under treatment with bicalutamide

Cellular PSMA expression is regulated by the androgen receptor, which is the target for the treatment of PC. Preclinical data indicate that PSMA expression is increased in CRPC [7] and under ADT [91]. In a first report, Hope et al. [92] indicated that an increased in vivo PSMA expression as imaged by ^68^Ga-PSMA-11 PET can be achieved in PC patients under ADT. The authors assumed that the effect seen in cell and animal models can be recapitulated in humans. Thus, it is near to suggest that ADT may increase the number of lesions visualized by PSMA-PET [91]. However, we have also seen the opposite of that in response to ADT with bicalutamide decreased ^68^Ga-PSMA-11 PET-uptake may be demonstrable after 1 week of treatment along with decreasing PSA values (Fig. 4). In fact, whereas Afshar-Oromieh reported a positive association with ADT [54, 55], others did not [66]. We assume that a better understanding of the temporal changes in PSMA expression is needed to leverage this effect for both improved diagnosis and possibly also for improved therapy as well as patient selection for therapy.

### Other factors associated with scan sensitivity

Other factors that have to be taken into account are the lack of PSMA over-expression on PC cells due to dedifferentiation [93], misinterpretation of positive lesions due to increased PSMA expression on other tumors or tumor neovasculature [94, 95], or the tumor sink-effect [96].

## ^68^Ga- or ^18^F–labeling for PC imaging?

Licensed ^68^Ge/^68^Ga generators are available in Europe and GMP-produced, with FDA-accepted quality, in the US. Dependent on demand, 1–2 generators will typically be required annually for local production. ^68^Ga has a half-life of 68 min and thus can only be shipped to close satellite centers. Recently, cyclotron-produced ^68^Ga has been introduced to potentially allow the production of larger quantities for centralized use [97]. In contrast, ^18^F has a half-life of 110 min and offers the possibility for using established satellite shipping infrastructure. The production demand for ^18^F is well scalable to adapt for the requested number of examinations. If necessary, also delayed imaging can be performed over a longer time. The positron energy of ^68^Ga is 1.90 MeV and its penetration depth is theoretically higher (lungs) but widely negligible in solid tissues using standard reconstruction algorithms and adjusted filtering. ^18^F has a positron energy of 0.65 MeV with theoretically higher resolution and also lower radiation burden. The labeling of ^68^Ga is done with chelator molecules, offering the possibility of kit-based formulations, whereas for ^18^F, prosthetic group molecules are necessary, which means hot cells, remotely controlled radiosynthesizers are due. ^68^Ga potentially offers an one-molecule theranostic approach, whereas ^18^F needs a tandem approach with a different chemical structure of diagnostic and a structurally related therapeutic tracer (e.g., PSMA-1007 / PSMA-617, DCFPyl / MIP-1095).

To date, the published experience with ^18^F–labeled PSMA-ligands is limited to about 100 patients. There are two potential radiolabeled ligands, ^18^F–DCFPyL [98–100] and ^18^F–PSMA-1007 [101, 102].

In a direct comparative study ^18^F–DCFPyL was compared to ^68^Ga-PSMA-11 in 25 patients with BR; another 62 patients underwent ^18^F–DCFPyL and 129 patients ^68^Ga-PSMA-11 PET [100]. The distribution pattern of both tracers was strongly comparable. However, in 36% of PSMA-positive patients, additional lesions on ^18^F–DCFPyL scan were observed. The authors suggested similar performance of ^18^F–DCFPyL and that the ^18^F–labeled ligand may even exhibit improved sensitivity in localizing relapse after RP for moderately increased PSA levels. Sensitivity increased abruptly when PSA values exceeded 0.5 μg/l. For PSA 0.5–3.5 μg/l, the sensitivity was 15/17 (88%) for ^18^F–DCFPyL and 23/35 (66%) for ^68^Ga-PSMA-11. Although the standard acquisition protocols, used for ^18^F–DCFPyL and ^68^Ga-PSMA-HBED-CC in this study, stipulate different activity and tracer uptake times after injection, the findings provide a promising rationale for validation of ^18^F–DCFPyL in future prospective trials. Furthermore, initial observation also indicates that ^18^F–DCFPyL is superior to conventional imaging modalities [103].

^18^F–PSMA-1007 is a promising alternative to ^68^Ga-PSMA-11 for diagnostic purposes as ^18^F–PSMA-1007 and ^177^Lu-PSMA-617 seem to be a perfect theranostic tandem [101]. In ten patients with biopsy-confirmed high-risk PC ^18^F–PSMA-1007 PET/CT had a NPV of 68% and an accuracy of 75%, while additional mpMRI in nine patients resulted in a NPV of 88% and an accuracy of 73% for total agreement. Near total agreement analysis resulted in a NPV of 91% and an accuracy of 93% for PET/CT and of 90 and 87% for mpMRI, retrospectively.

## New developments

Recent studies have demonstrated the potential of ^64^Cu-labeled PSMA-617 ligand in patients with recurrent disease and in selected patients for primary staging with progressive local disease. Grubmüller et al. [37] detected a positive PC tumor binding in 23/29 patients, with the salivary glands, kidneys, and liver showing the highest tracer uptake. Moreover, Cantiello et al. [104] showed for 23 patients with intermediate to high-risk PC a sensitivity of 87.5% and a specificity of 100% for primary LN staging at 4-h postinjection before RP. ^64^Cu allows the concept for satellite distribution to clinical PET centers that lack radiochemistry facilities for the preparation of ^68^Ga-PSMA ligand due to longer-lived positron emitter with good image quality. ^64^Cu-PSMA-617 may also offer the possibility of pre-therapeutic dosimetry in the theranostic approach. Tables 2, 3 and 4 and Fig. 2 list the current available data on ^68^Ga-PSMA PET/CT results.

**Table 2:** ^68^Ga-PSMA PET/CT: Summary of imaging results - status October 2017

PRIMARY STAGING		SECONDARY STAGING	
Sachpekidis et al. [39] *n* = 24	Increased tracer accumulation with time	Ceci et al. [50] *n* = 70	PSA_dt_ 6.5 months & PSA 8.8 ng/ml are cut-off values for PET positivity
Uprimny et al. [40] *n* = 90	Detection rate is dependent on GS and PSA level	Verburg et al. [51] *n* = 155	PET positivity: PSA levels and shorter PSA_dt_ are independent predictors
Maurer et al. [41] *n* = 130 Maurer et al. [67] *n* = 92 Maurer et al. [75] *n* = 332	Superior detection rate compared to CT/mpMRI in high- to intermediate-risk patients Superiority to conventional imaging proved by histopathology PET/CT < PET/MR	Afshar-Oromieh et al. [54] *n* = 319 Afshar-Oromieh et al. [55] *n* = 1007 Afshar-Oromieh et al. [86], *n* = 112	Positivity correlates with PSA level and ADT but not with PSAdt and GS Increased detection by additional late imaging at 3 h p.i.
Eiber et al. [66] *n* = 53	Superiority of PET mpMRI over mpMRI or PET alone but no correlation with GS and PSA value	Eiber et al. [56] *n* = 248 Einspieler et al. [57] *n* = 118	Scan positivity correlates with GS but not with ADT Superiority to CT Detection rate correlates with PSA level and concomitant ADT
Giesel et al. [68] *n* = 10 Zamboglou [69, 70] *n* = 22	PET/CT and mpMRI correlate with tumor allocation proven by histopathology	Morigi et al. [85] *n* = 38	Superiority over ^18^F–cholin Management impact in 63% of patients
Budäus et al. [42] *n* = 30	LN detection rate is determined by LN size as proven by pathohistology	Pfister et al. [80] *n* = 28	Superior detection of local recurrence and/or metastases
van Leeuwen et al. [44] *n* = 30	LN detection rate is dependent on LN size	van Leeuwen et al. [53] *n* = 70 (PSA 0.05–0.1 ng/ml)	Management change in 28.6% of patients with impact on changes in RT-volume
Herlemann et al. [45] *n* = 20	Increased sensitivity of PSMA-PET to CT proven by histopathology	Herlemann et al. [45] *n* = 14	Increased sensitivity of PSMA-PET to CT
Demirkol et al. [46] *n* = 8	Increased sensitivity of PSMA-PET to CT	Kabasakal et al. [58] *n* = 50	PET positivity correlates with PSA level and GS
Sterzing et al. [47] *n* = 15	increased sensitivity of PSMA-PET to CT	Sachpekidis et al. [59] *n* = 31	Positivity correlates with PSA level; increasing uptake during dynamic PET acquisition
Sahlmann et al. [48] *n* = 12	Increased detection with late imaging under furosemide	Rauscher et al. [61] *n* = 48	Superior to conventional imaging proved by histopathology
Iagaru et al. [71] *n* = ongoing prospective study	of PET/mpMRI in intermediate- and high-risk patients	Kranzbühler et al. [72] *n* = 56	Superiority of PET/mpMRI over PET/CT for local recurrence
Giesel et al. [38] *n* = ongoing prospective study	Ongoing prospective study of PET/CT in intermediate and high-risk patients	Freitag et al. [73, 74] *n* = 119	Superiority of PET/mpMRI over PET/CT for local recurrence but not for distant metastases
		Schmuck et al. [87] *n* = 184	In 5.4% of patients increased detection rate with delayed imaging
		Uprimny et al. [88] *n* = 80	In 9.4% increased detection rate by dynamic imaging

**Table 3:** Efficiency of ^68^Ga-PSMA PET/CT in patients with biochemical relapse (low rising PSA) – status October 2017

AUTHORS	DESIGN	TOTAL number	PSA 0.0–0.1	PSA 0.1–0.2	PSA 0.2–0.3	PSA 0.3–0.5	PSA 0.5–0.8	PSA 0.8 < 1	PSA 1–2	PSA > 2	PET/CT SCANNER	IMAGE AQUISITION
van Leeuwen et al. [53]	Prosp	*n* = 300	*n* = 13	*n* = 22	*n* = 17	*n* = 11	*n* = 7				Ingenuity TOF, Philips	45 min
			8%	23%	58%	36%	57%					
Afshar-Oromieh et al. [54] Afshar-Oromieh et al. [55]	Retrosp Retrosp	*n* = 319 (83%)* *n* = 801/ 1007 (79.5%)*	*n* = 8/17		*n* = 5/10		*n* = 14/24		*n* = 28/39		Biograph, Siemens	60 min
			47.10%		50%		58.30%		71.80%			
			*n* = 32/69		*n* = 50/108		*n* = 87/119		*n* = 132/166	*n* = 467/509		
			46%		46%		73%		80%	91.75%		
Eiber et al. [56] Maurer et al. [75] Einspieler et al. [57]	Retrosp Retrosp Retrosp	*n* = 248 (90%)* *n* = 332 107/118 (90.7%)*			*n* = 11/19		*n* = 24/33		*n* = 67/72	*n* = 120/124	Biograph, Siemens	60 min
					57.89%		72.73%		93.06%	96.77%		
					*n* = 13/23		*n* = 25/35		*n* = 67/71	*n* = 122/127		
					56.5%		71.4%		94.4%	96.1%		
										*n* = 107/118		
										(90.7%)		
Verburg et al. [51]	Retrosp	*n* = 155 (89%)*	*n* = 12/27						*n* = 15/19	*n* = 97/109	GEMINI TF16, Philips	60 min
			44%						79%	89%		
Morigi et al. [85]	Prosp	*n* = 37/38	*n* = 8/16				*n* = 10/14			*n* = 7/8	Ingenuity TOF, Philips	45 min
			50%				71%			88%		
Kabasakal [58]	Retrosp	*n* = 29/50 (58%)*	*n* = 4		*n* = 11					*n* = 14	Biograph, Siemens	45–60 min
			33%		50%					87.5%		
Ceci et al. [50]	Retrosp	*n* = 52/70 (74.2%)*	*n* = 21				*n* = 49				Discovery, GE	8 min, 60 min, (120 min)
			47.60%				85.70%					
Sachpekidis et al. [59]	Retrosp	*n* = 22/31 (71%)*	*n* = 4/11						*n* = 15/20		Biograph, Siemens	Dynamic for 60 min (pelvis), WB at 80–90 min p.i.
			36.36%						75%			
Albisinni et al. [62]	Retrosp	*n* = 98/131 (75%)	45%						83%		GE Discovery 690	60 min

*Overall detection rate

**Table 4:** Overview of treatment results, side-effects, and quality of life – status October 2017

Authors	Substance	Number of patients	Therapy Scheme	PSA RESPONSE		RECIST/PERCIST RESPONSE						Quality of life	Side effects		
				Any decrease	≥50% decrease	CR	PR	SD	PD	PFS	OS		Nephrotoxicity	Hematotoxicity	Xerostomia/xerophthalmia
Baum et al. [111]	PSMA-I&T	56 25	3.4–8.7 GBq /cycle (> 2–5 cycles)	45/56 80.4%	25/56 58.9%		14	2	9	13.7 months	Not reached	VAS score: Pain reduction in 2/6 patients, improvement in KPS	None	Insignificant decreases of erythrocytes and leucocytes – but no grade 3 or 4	Two transient mild cases after 3 and 4 cycles. (8%)
Kulkarni et al. [112]	PSMA-617 PSMA-I&T	117	6 (2–9.7 GBq)/cycle 1–7 cycles	61/80 76.3%	46/87 57.6%	5/58 8.6%	12/58 20.7%	29/58 39.7%	18/59 31%	10.7 months		pain reduction and quality of life improved significantly in symptomatic patients	No grade 3 to 4	No grade 3 to 4	Five cases of mild dryness (4.2%), frequent fatigue
Rahbar et al. [113]	PSMA-617	145	5.8 GBq (2–8 GBq)/cycle 1 cycle 2 cycles 3 cycles 4 cycles	65/99;66% 44/61;57%	40/99;4035/61;57 13/20;653/3;100	2%	45%	25%	28%			n.a.	n.a.	18/145 patients grade 3–4 Grade 3–4: anemia 10% thrombocytopenia (4%) leukopenia (3%)	8%
Rahbar et al. [114]	PSMA-617	74 23/74	5.9 ± 0.5 GBq (1 cycle)	23/74 (31%)	n.a.	n.a.	n.a.	n.a.	n.a.	n.a.	n.a.	n.a.	Grade 0–1	n.a.	n.a.
Yadav et al. [115]	PSMA-617	31	5 ± 1.8 MBq (1–4 cycles)	22/31 70.9%	n.a.	2/6	3/6	1/6	6/31	12 months	16 months	ECOG 3➔1 VAS-Score 9 ➔ 1 KPS 40 ➔ 80	No grade 3 or 4		
Heck et al. [116]	PSMA-I&T	19 10/19	7.4 GBq/cycle (1–4 cycles) 3–4 cycles	10/18 56%	8/18 44%	1/19 5% 1/10	n.a.	12 63% 6/10	6 32% 3/10	n.a.	n.a.	bone pain reduction in 85%, 74% ECOG-improvement	No grade 3–4		Dry mouth 7/19 (37%)
Fendler et al. [117]	PSMA-617	15	3.7 GBq (*n* = 5) 6 GBq (*n* = 10) 2 cycles	12/15 (80%)	9/15 > 50%		4/15 27%	6/15 40%	5/15 33%			9/15 QoL improvement 7/10 Pain relief	No grade 4		
Scarpa et al. [118]	PSMA-617	10	(5.4–6.5 GBq)/cycle 3 cycles	3/10 (33%)	n.a.		3/10	1/10	1/10			n.a., 3 patients showed mixed response	No grade 3 to 4		
Kratochwil et al. [119]	PSMA-617	30 11	3.7–6.0 GBq /cycle (1–3 cycles) 3 cycles	21/30	13/30 8/11	6 patients, 50% decreased SUV_max_						No data	No acute and late effects up to 24 weeks	Leukopenia: Grade 2: 2 patients thrombocytopenia: 1 patient changed from Grade 2 to Grade 3. After 3 cycles decreased platelets (−20%) at 24 weeks	2/30 after the third cycle
Ahmadzadehfar et al. [120]		22/24	4.1–7.1 GBq/cycle 2 cycles	68.2%		n.a.	n.a.	n.a.	n.a.	n.a.	n.a.	n.a.	No grade 3 or 4	2 patients: Grade 2–3 anemia	Dry mouth in 8.7%
Ahmadzadehfar et al. [121]		52	6 (4.0–7.2) GBq/cycle 1 cycle 2 cycles 3 cycles	42; 80.8% 35; 67.3% 28;53.8%	23;44.2% 12; 3.1% 10; 19.2%	n.a.	n.a.	n.a.	n.a.	60 weeks	n.a.	n.a.	n.a.	n.a.	n.a.
Yordanova et al. [122]	PSMA-617	55	6 GBq (4.0–7.1 GBq) > − 3 cycles		n.a.	n.a.	n.a.	n.a.	n.a.	n.a.	n.a.	n.a.	No grade 3–4	n.a.	n.a.
Bräuer et al. [123]	PSMA-617	59	5.9–6.3 GBq/cycle 3 cycles (1–7 cycles)	91%	53%	n.a.	n.a.	n.a.	n.a.	18 weeks	32 weeks	Transient fatigue in 12 patients	No grade 3 to 4	2 patients grade 3 leucopenia and thrombocytopenia (3%), grade 3 anemia in 11 patients (19%)	15 patients (25%) xerostomia, 1 patient mild dryness of the eyes

*VAS score* Visual Analogue Scale, *KPS* Karnofsky Performance Score, *ECOG* toxicity and response criteria of the Eastern Cooperative Oncology Group

## PSMA-directed radioligand therapy (PRLT) – Theranostic concept of personalized therapy

Initially, almost all patients with hormone-naive PC have a good response to the well-established anti-androgen treatments. Over the last several years, even for patients with CRPC, significant improvements were observed following treatment with the androgen-receptor antagonist enzalutamide or the CYP17A1-inhibitor abiraterone [110]. However, resistance to these treatments occurs frequently within 1 to 2 years. For this reason, a targeted radionuclide approach could be an attractive therapy option. The PSMA-targeting theranostic concept potentially offers advantages not only in regard to diagnosis but also the therapy of CRPC patients, if labeled with ^177^Lu [111–124], ^131^I [125, 126], Auger [127], or an alpha-emitting isotope [128–130].

So far, most patients received theranostics for PC under compassionate use conditions according to the Declaration of Helsinki [131] after treatment failure following chemotherapy, monoclonal antibody therapy, hormonal therapy, or ^223^Ra-chloride therapy receiving PRLT as an ultimate treatment option. As a matter of fact, so far, centers reporting data on PRLT have been well established with peptide receptor radionuclide therapy (PRRT) in neuroendocrine tumors in the past. Usually, the precursors are commercially obtained, labeled with the radionuclide in specified radiochemical laboratories, and applied to patients using similar conditions as with radiolabeled somatostatin analogues. Hereto, fractionation of the dose applied to the patient was a prerequisite of the treatment scheme and dosimetry mandatory as well as follow-up of the patient by ^68^Ga-PSMA-directed PET/CT or PET/mpMRI using the PERCIST criteria.

## ^177^Lu-PSMA-ligands

### Dosimetry and side effects

Due to substantial individual variance, dosimetry is mandatory for a patient-specific approach following ^177^Lu-PSMA-617 therapy [118]. Following therapy with an accumulated activity of 18.2 ± 0.9 GBq, the mean absorbed tumor dose amounted to 2.8 ± 0.52 Gy/GBq, the kidney dose to 0.6 ± 0.36 Gy/GBq, and the red bone marrow dose activity to 0.04 ± 0.03 Gy/GBq. The mean dose to the parotid glands was 0.56 ± 0.25 Gy/GBq, to the submandibular glands 0.50 ± 0.15 Gy/GBq, to the lacrimal glands 1.01 ± 0.69 Gy/GBq, and the mean effective dose was 0.08 ± 0.07 Sv/GBq (range, 0.02–0.26 Sv/GBq). Response to therapy was observed already after one or two treatment cycles in terms of decreased SUV_max_ values and PSA response despite no grade 3 to 4 toxicity. Thus, Scarpa et al. [118] concluded that higher activities and/or shorter treatment intervals should be applied and that a total activity of 30 GBq given 6 to 10 weeks apart is safe, especially considering the dose limit to the kidney and bone marrow. Large inter-individual variation and the need for patient individual dosimetry was also postulated by Kabasakal et al. [132] who reported an absorbed kidney dose of 0.9 ± 0.40 Gy/GBq for ^177^Lu-PSMA-617. Yordanova et al. [122] reported no grade 3–4 nephrotoxicity, but found an elevation of cystatin C in 32/55 patients (58%) of whom 14 patients had elevated cystatin C before ^177^Lu-PSMA-617 treatment. Furthermore, a significant correlation of renal function was found for age (*p* < 0.05), hypertension (*p* = 0.001), and pre-existing kidney disease (*p* = 0.001). If the kidney-to-tumor ratio presents a problem due to prior therapy or presence of accompanying diseases diabetes and hypertension, the co-administration of PSMA inhibitors such as 2-(phosphonomethyl)penanedioic acid (2-PMPA) might be considered [133]. Okamoto et al. [134] performed dosimetry studies in 18 patients who had received 1–4 treatment cycles of ^177^Lu-PSMA-I&T showing that organ and tumor-absorbed dose were comparable to ^177^Lu-PSMA-617. Furthermore, they showed that the absorbed organ doses were relatively constant among the four different treatment cycles. Regarding the kidneys, also these authors suggested that a cumulative activity of 40 GBq would be safe and justifiable.

Transient xerostomia, which may impair quality of life, occurs in 5 to 10% of patients treated with ^177^Lu-PSMA ligands and seems to be caused by high uptake of the radiopharmaceutical in the salivary glands. Repeated cycles of ^177^Lu-PSMA-617 therapy led to significantly decreased SUV_max_ values on ^68^Ga-PSMA-11 PET/CT accompanied by significant volume reduction (*p* < 0.05) of the salivary glands [118]. The frequently used cool bags during administration of the radioactivity may help to reduce PSMA ligand uptake, as there is evidence of reduced ^68^Ga-PSMA-11 uptake in the glands in terms of decreasing SUV_max_ values when cooled [135]. Dysfunction is usually transient and a maximal dose limit of 45 Gy has been suggested with a dose of 30 Gy for total recovery within 2 years [136]. Assuming an absorbed dose of around 0.5–1.0 Gy/GBq for the salivary glands, the mean absorbed dose amounts to around 10 Gy when an activity of 18 GBq is administered, suggesting that an accumulated total activity of 50 GBq of ^177^Lu-PSMA-617 could be administered with large inter-individual variation.

The risk of development of hematotoxicity is increased in extensively pretreated CRPC patients. Especially patients with extensive bone marrow involvement and previous chemotherapies may respond with higher hematotoxicity. To decrease the probability of severe bone marrow toxicity, a threshold of 2 Gy absorbed dose to the red marrow is generally recommended in radionuclide therapy dosimetry [137]. The mean red marrow dose amounts to around 0.04 Gy/GBq, which results in an absorbed dose of 0.7 Gy [118], suggesting that the tolerable accumulated activity for the bone marrow lies around 45 GBq of ^177^Lu-PSMA-617, again by large inter-individual variation, indicating the importance of pre-therapeutic dosimetry. Kabasakal et al. [132] suggested that even an activity of 65 GBq of ^177^Lu-PSMA-617 is clinically safe for the bone marrow. Reported differences may lie in different patient population and selection for therapy. For instance, the Bad Berka group [111] reported no grade 3 or 4 side effects, whereas the Heidelberg group [119] and also the German multi-center study [113] had some grade 3 and 4 toxicities. We believe that fractionation of the activities is the best way to avoid severe bone marrow toxicity as published tolerance limits do not seem to be reliable for the concept.

### Response to therapy

It can be assumed that the strong tumor response is attributable to the high doses delivered to the tumors (Table 4). The absorbed tumor dose amounts to > 50 Gy in patients receiving an accumulated mean activity of 18 GBq [118], but may also come up to 500 Gy [111] for small LN metastases. Generally, the absorbed tumor dose is about ten times higher than the dose calculated for the critical organs kidney and salivary glands [117, 118, 132, 133]. The potential of the ^68^Ga/^177^Lu-theranostic concept was first proven using the ligands PSMA-I&T [24] and PSMA-617 [138]. Initial reports suggest that pretherapeutic PET data in terms of SUV_max_ values correlate with absorbed tumor dose and changes in terms of decreasing SUV_max_ values in patients receiving either ^177^Lu-PSMA-I&T [134] or ^177^Lu-PSMA-617 [118], emphasizing also the need of PSMA ligand PET imaging for patient selection.

**Fig. 5:**
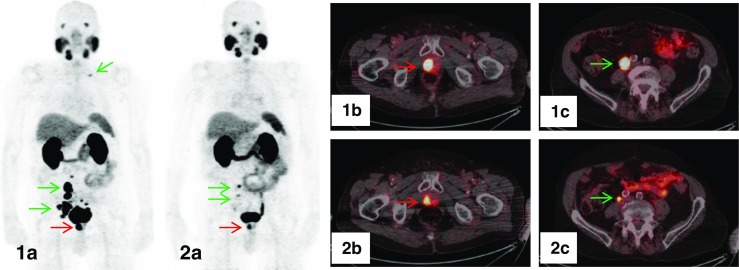
Follow-up ^68^Ga-PSMA-11 PET/CT (low-dose CT) of an 80-year-old CRPC-patient who had received treatment with ^177^Lu-PSMA-617. On the PET scan prior to therapy, local tumor in the prostate bed (*red arrow*), multiple abdominopelvic and one cervical LN metastases (*green arrows*) were clearly visible on maximum intensity projection (MIP) (1a) and on fused axial PET/CT-images (1b, 1c). Restaging PET/CT performed 8 weeks after administration of four cycles of ^177^Lu-PSMA-617 with a total accumulated activity of 24.9 GBq showed a significant reduction of the primary tumor (*red arrow*) and an impressive partial response of LN metastases with only small metastases left in the right iliac region (*green arrows*), as displayed on MIP (2a) and fused axial PET/CT-images (2b, 2c)

Response to PRLT is usually assessed in terms of biochemical response by decreasing PSA values following each therapy cycle (Fig. 5). Responding patients usually show a PSA decline already after one therapy cycle with 6 GBq only. So far, only a few studies reported response assessment using the RECIST or PERCIST criteria [111, 112, 115–117], and also clinical response data are limited [111, 112, 115–117]. In addition, response assessment is difficult as well, as patients may respond remarkably to PRLT at one metastatic lesion site but may develop new lesions at another site, thereby showing a mixed response [MX; 123]. Especially bone metastases and small LN metastases may escape detection by stand-alone conventional imaging, demanding PET follow-up.

Using ^177^Lu-PSMA-I&T Baum et al. [111] were the first who reported on a larger series of patients who received up to six therapy cycles (3.4–8.7 GBq/cycle) given approximately 8 weeks apart. Overall, a decreased PSA response was found in 45/56 (80.4%) of patients. A PSA decline of ≥50% was seen in 33 patients (58.9%). In 25 patients who received more than two therapy cycles, a partial remission (PR) was reported in 14, a stable disease (SD) in two, and progressive disease (PD) in nine patients by ^68^Ga-PSMA-11 PET/CT. The median PFS was 13.7 months, and the median OS was not reached during the follow-up period of 28 months. Additionally, significant improvement of clinical symptoms and excellent pain palliation can be achieved as reported by the same authors later on when they summarized their results in 190 patients receiving 1–7 treatment cycles with a median activity of 6 GBq per cycle [112]. In 80 patients who had at least one course of PRLT with either PSMA-617 or PSMA-I&T, a PSA reduction ≥50% was seen in 46/80 (57.6%), any level of  PSA reduction in 61/80 (76.3%) patients. In 58 patients, the response according to RECIST was as follows: complete remission (CR) in five patients (8.6%), PR in 12 patients (20.7%), SD in 23 patients (39.7%), and PD in 18 patients (31.0%). In general, the authors reported that LN metastases responded better than bone metastases. Survival data were analyzed in 104 patients and showed a progression-free survival (PFS) of 10.7 months from the commencement of therapy. The most common adverse event was mild fatigue and five patients (4.2%) reported mild dryness of the mouth.

Recently, the first retrospective multicenter study from 12 German institutions was published by Rahbar et al. [113]. The institutions used ^177^Lu-PSMA-617 and reported the results from 145 patients who received 1–4 cycles (in total 248 cycles), 8–12 weeks apart, 2–8 GBq ^177^Lu-PSMA-617. The median follow-up time was 16 weeks (range, 2–30 weeks). The primary end point for efficacy was biochemical response defined by a PSA-decline ≥50% from baseline at least 2 weeks after the start of PRLT. The patient characteristics included a broad range of previous therapies such as ADT, chemotherapy, abiraterone, enzalutamide, ^223^Ra or RT. The administered activities also had a broad range, but most patients received one or two cycles only. The overall biochemical response rate was 45% after all therapy cycles, whereas 40% of the patients already responded after the first therapy cycle. The secondary endpoint was investigator-assessed conventional imaging response showing CR in 2%, PR in 45%, SD in 28%, and PD in 25%. In terms of toxicity, hematotoxicity grade 3–4 occurred in 18 patients with 3% leukopenia, 10% anemia, and 4% thrombocytopenia and xerostomia was reported in 8% of patients. Noteworthy, elevated alkaline phosphatase (AP) and the presence of visceral metastases were negative predictors, whereas the total number of therapy cycles was a positive predictor of biological response.

Yadav et al. [115] treated 31 patients with ^177^Lu-PSMA-617 with 1–4 cycles, the average activity was 5 ± 1.8 GBq. Biochemical response was observed in 22/31 patients (70.9%), metabolic response according to PERCIST criteria showed CR in 2/6 patients, PR in 3/6 patients, SD in 1/6 patients, and clinical response measured by Visual Analogue Scale (VAS) score, analgesic score (AS), Karnofsky Performance Status (KPS), and toxicity and response criteria of the Eastern Cooperative Oncology Group (ECOG) performance status improved in approximately two-thirds of the patients.

In 19 patients, Heck et al. [116] used ^177^Lu-PSMA-I&T administering an activity of 7.4 GBq/cycle. Combined assessment of bone and soft tissue metastases showed a CR in 5% of patients, SD in 63% and PD in 32%, while ECOG performance status improved or was stable in 74% and pain reduction was seen in 58% of patients.

Fendler et al. [117] reported PR in 4/15, SD in 6/15, and PD 5/15 using RECIST criteria after 2 PRLT-cycles with ^177^Lu-PSMA-617. Furthermore, significant pain relief was documented in 7/10 symptomatic patients and improvement of Quality of Life (QoL) in 9/15 patients.

Scarpa et al. reported in 5/10 consecutive patients PSA response who also showed an objective radiological and metabolic response by ^68^Ga-PSMA-11-PET/CT in terms of PR, MX, or SD [118].

Using PSA as response parameter, Kratochwil et al. [119] found in eight of 11 patients who were treated with three cycles of ^177^Lu-PSMA-617 a sustained PSA-response (> 50%) for over 24 weeks, which correlated with radiological response. PSA response can be seen as early as after one therapy cycle only with decline of more than 50% from baseline values [120]. In 47/74 patients (64%), a PSA decline was noticed after one therapy cycle only (5.9 ± 0.5 GBq) with a pronounced decline of > 50% in 23/74 (31%) of patients [114]. Similar response with a PSA decline > 50% in about 60% of patients was reported by Baum et al. [111] receiving up to five cycles of ^177^Lu-PSMA-I&T. In accordance with the findings of Kratochwil [119], also Scarpa et al. [118] found diverging results of PSA levels and PET/CT as well as whole-body imaging questioning PSA as a reliable parameter for tumor response evaluation.

Ahmadzadehfar et al. [120, 121, 139] reported in about 70% of patients a response to ^177^Lu-PSMA-617 treatment with PSA decline. In their most recent retrospective evaluation [121, 122], the authors showed a ≥ 50% PSA decrease in 44.2% after the first, 23.1% after the second, and 19.2% after the third therapy cycle with around 6 GBq each. The median OS was significantly longer for patients who responded with any PSA decline compared to patients without PSA decline (68 vs. 33 weeks). Noteworthy, patients who showed no PSA response after the first cycle responded after the second or third therapy cycle. Regarding side effects, only two patients developed grade 3 anemia, while no severe nephrotoxicity was reported. No detailed conclusion on salivary gland toxicity can be drawn from their publications.

Bräuer et al. [123], who retrospectively reviewed 59 patients, calculated that a PSA decline after the first treatment cycle was associated with a longer OS. In their study, the median estimated PSA-PFS was 18 weeks and only AP < 220 U/l was significantly associated with a longer PFS.

### Alpha-emitting PSMA ligands

Recently, first-in-human treatment with an α-emitting PSMA-ligand was presented by Kratochwil et al. [129]. Salvage therapies empirically conducted with 50 kBq/kg (*n* = 4), 100 kBq/kg (*n* = 4), 150 kBq/kg (*n* = 2), 200 kBq/kg (*n* = 4) of ^225^Ac-PSMA-617 were evaluated retrospectively regarding toxicity and treatment response. Eight out of 14 patients received further cycles in either 2- or 4-month intervals with identical or de-escalated activities. Dosimetry estimates for 1 MBq of ^225^Ac-PSMA-617 assuming a relative biological effectiveness of 5 revealed mean activities of 2.3 Sv for salivary glands, 0.7 Sv for kidneys, and 0.05 Sv for red marrow that are composed of 99.4% alpha, 0.5% beta, and 0.1% photon radiation, respectively. In clinical application, severe xerostomia became the dose-limiting toxicity if treatment activity exceeded 100 kBq/kg per cycle. At 100 kBq/kg duration of PSA-decline was < 4 months, but if therapy was repeated every 2 months, patients experienced additive anti-tumor effects. Treatment activities of 50 kBq/kg were without toxicity but induced insufficient anti-tumor response in these high-tumor-burden patients. Remarkable anti-tumor activity by means of objective radiological response or PSA decline was observed in 9/11 evaluable patients. The authors concluded that for advanced-stage patients, a treatment activity of 100 kBq/kg ^225^Ac-PSMA-617 per cycle repeated every 8 weeks presents a reasonable trade-off between toxicity and biochemical response. The dosimetry estimate using increasing dose of ^225^Ac-PSMA-617 targeted α-therapy with ^225^Ac-PSMA-617, although still experimental, obviously has strong potential to significantly benefit advanced-stage PC patients.

Sathekge et al. [130] reported a first-in-human treatment of ^213^Bi-PSMA-617-targeted PRLT and presented impressive preliminary response in one patient after two cycles with a cumulative activity of 592 MBq.

### Iodinated ligands

Afshar-Oromieh et al. [126] used ^131^I–MIP-1095 and showed that the first dose of PRLT presented with low side effects and could significantly reduce the tumor burden in the majority of patients. The second and third therapies were less effective and presented with more frequent and more intense side effects, especially hematologic toxicities and xerostomia. A preliminary evaluation by Tesson et al. [125] using ^131^I–MIP-1095 in combination with radiosensitizing chemotherapeutic drugs increased tumor uptake in PC cells and may thus optimize PRLT when combined.

### Future of PRLT

Tailored multimodality options may become the future of “prosta(g)nostic” concepts including the combination of existing therapies such as with ^223^Ra, RT, and probe-radioguided surgery. Future phase II/III studies are warranted to elucidate the survival benefit of PRLT in patients with CRPC. The major limitations of existing retrospective PRLT reports are heterogeneous patient cohorts suffering from very advanced disease stages with negative prognostic factors such as GS, visceral metastases, and extensive pre-treatments (negative referral bias). Results might be better in CRPC patients with confined disease extent and fewer pre-treatments. PRLT could better be given earlier and eventually in combination with new-generation ADT such as enzalutamide with acceptable side effects and improved QoL.

In fact, van Eyben et al. [140] systematically analyzed PRLT with ^177^Lu-PSMA-617 and ^177^Lu-PSMA-I&T against third-line treatment of PC with abiraterone, enzalutamide, and cabazitaxel. Ten studies consisting of 746 patients reported third-line treatment with abiraterone, enzalutamide, and cabazitaxel. Combined, 20% of the patients had ≥50% reduction of PSA. Ten studies consisting of 506 patients reported ^177^Lu-PSMA PRLT. Forty-two percent of the patients had ≥50% reduction of PSA. Third-line treatment gave a lower rate of ≥50% reduction of PSA than ^177^Lu-PSMA PRLT (*p* < 0.001, χ2 test). This analysis indicates that RLT can be more effective than established therapies.

Definition of response to PRLT is a problem. Treatment stratification is based on PET/CT positivity and disease progression thereby tailoring patient selection for PRLT. Only a combination of available measures (clinical status, quality-of-life measurements, PSA-values, PET/CT with PSMA-ligands, CT, MR) seems to be appropriate, the most important one probably remains the clinical status of the mostly elderly patient. Increasing the treatment activity or number of cycles, or shortening the time interval between the cycles are options that may be considered in future prospective trials [118]. The question of the necessity of kidney protection seems to be best answered on individual kidney function as a result of pre-treatments or existing concomitant diseases, i.e., diabetes and hypertension [133]. A future possibility would be the combination of radiosensitizing chemotherapeutic drugs with PRLT [125]. Kulkarni et al. [112] suggested that “*the ideal patient for PRLT could possibly be one receiving PRLT before chemotherapy (or maybe in combination with chemotherapy) with good baseline bone marrow function and a good baseline performance status*”.

Syngeneic models of murine PC may be useful for studying the effects of PSMA-directed PRLT combined with potentially synergistic pharmacological approaches [141] as the rapid clinical translation of theranostics of prostate cancer (“prostanostics”) lacks optimization of the radiation dose.

Results with α-emitter-PSMA-PRLT are preliminary [129, 130]—though very encouraging—but potential side effects need to be better understood.

The existing dosimetry data suggest that a combination of ^223^Ra and ^177^Lu-PSMA-617 may evolve into a tailored treatment strategy for advanced-stage CRCP patients. ^223^Ra is readily available and clinically effective for skeletal metastases [18, 142], which do seem to respond to ^177^Lu-PSMA PRLT heterogeneously. Furthermore, side effects for ^223^Ra-therapy are moderate [143]. Hematotoxicity following treatment with ^223^Ra (3.5 MBq per injection) was calculated to range from 0.5 Gy [144] to 1.5 Gy [145]. Lassmann and Nosske [146] reported 1.3 Gy using biokinetic models based on ICRP guidelines [147]. In fact, Ahmadzadehfar et al. [120] recently have shown that repeated cycles of ^177^Lu-PSMA-617 PRLT after previous ^223^Ra-therapy are safe, with only a very small probability of hematotoxicity. Practically, α-particles have clear advantages over ß-particles for treatment of micrometastases including a short tissue range, which delivers circumscribed energy deposition while sparing surrounding normal tissue [148]. Thus, side effects following ^223^Ra-therapy mainly stem from the bone marrow, whereas ^177^Lu-PSMA-617-therapy practically shows little negative effect on bone marrow. In order to treat LN and visceral metastases, it would seem appropriate to add ^177^Lu-PSMA-617 to an existing ^223^Ra-therapy as side effects to the salivary glands from ^177^Lu-PSMA-617, are mostly transient or relieved.

## Conclusions

**Fig. 6:**
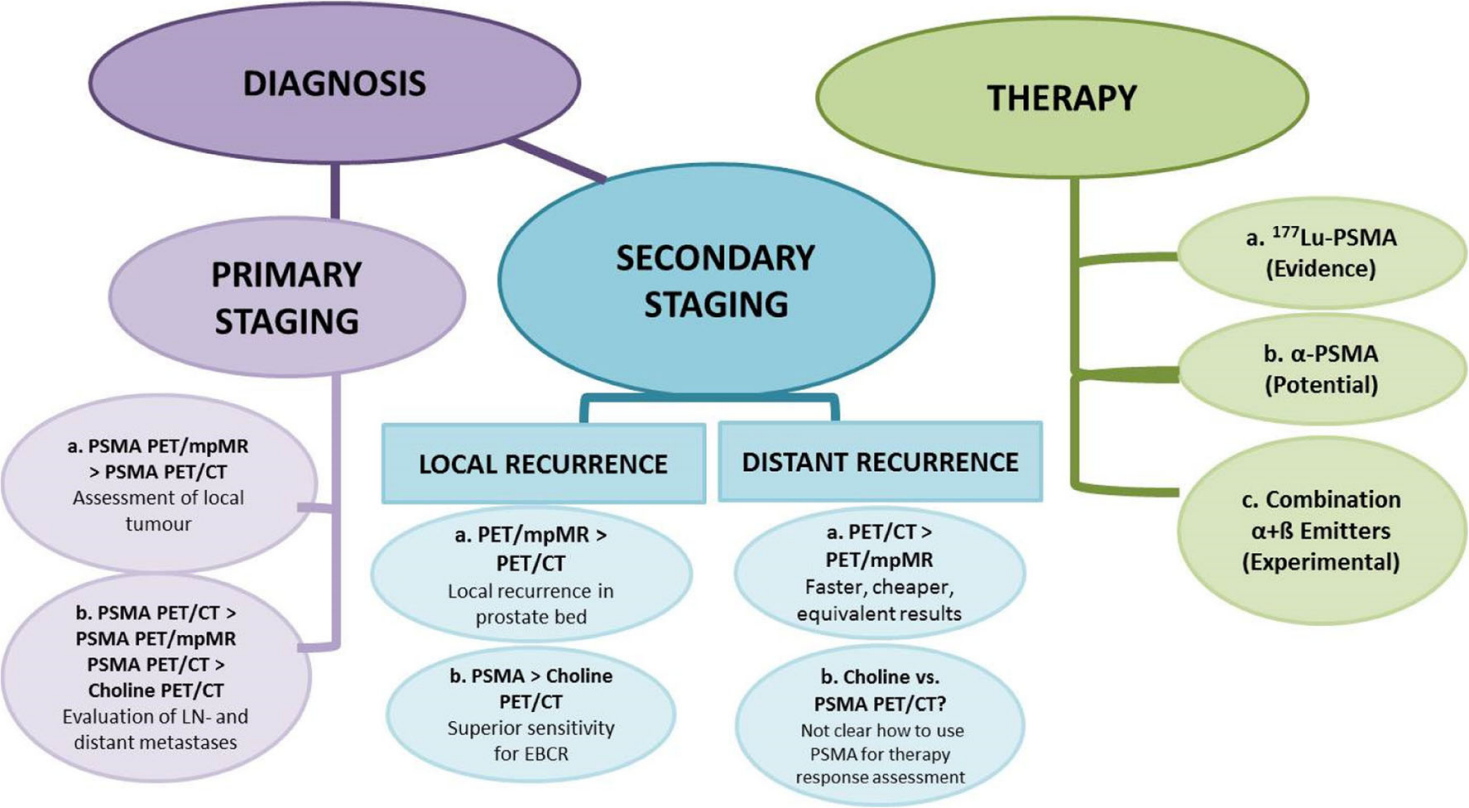
Current status of theranostics in prostate cancer ("Prostanostics"). The current status of PSMA-directed theranostics in PC patients is based on retrospective studies. ^68^Ga-PSMA PET/CT in primary staging is meaningful in patients with high-risk PC for local tumor assessment. The combination with pelvic multi parametric (mp)MR (or PET/mpMR) reaches the highest impact on patient management. In secondary staging for local recurrence, ^68^Ga-PSMA PET/mpMR is superior to PET/CT, whereas for distant recurrence, PET/CT has equivalent results and is faster and cheaper compared to PET/mpMR. ^68^Ga-PSMA PET/CT is superior to ^18^F / ^11^choline PET/CT in primary staging as well as in secondary staging. Significant clinical results have so far been achieved with the subsequent use of radiolabeled PSMA ligands in the treatment of CRPC, especially with ^177^Lu-PSMA ligands. Potential results have been demonstrated for α-emitting ligands and the combination treatment of β- and α-emitters is discussed

The current status of theranostics in prostate cancer (“Prostanostics”) is summarized in Fig. 6.

### Imaging

In high-risk PC patients, the likelihood of LN and bone metastases is increased and these can be detected by ^68^Ga-PSMA-ligand PET/CT or PET/MR. Several studies demonstrate the superiority of ^68^Ga-PSMA PET/CT over CT, mpMRI, bone scan alone / or over ^18^F–choline PET/CT, for detection of metastases for initial staging at primary diagnosis. This can significantly influence patient management, although the impact on overall survival remains unanswered. There may be a role for ^68^Ga-PSMA-PET in PC patients with an intermediate risk also.

^68^Ga-PSMA ligand PET/CT or PET/MR has a high detection rate including low biochemical PSA recurrence with significant impact on patient management in terms of therapy response assessment, selection of patients for salvage therapy, or targeted biopsy. In principal, PET positivity correlates to increasing PSA values with a 50% of chance to detect a tumor lesion when PSA = 0.5 ng/ml and ≥90% when PSA = 1.0 ng/ml, which basically shows that PSMA PET has a superior sensitivity to ^18^F / ^11^C–choline. For local recurrence, ^68^Ga-PSMA PET/MR or PET/CT in combination with mpMR is most appropriate. Distant recurrence can be imaged best by PET/CT, which is faster and cheaper compared to PET/MR and has a similar accuracy. The availability from ^68^Ge/^68^Ga generators favors the use of ^68^Ga-PSMA ligand PET/CT by institutions that do not have access to a cyclotron as ^18^F–PSMA ligand imaging is emerging.

Prospective multicenter diagnostic trials are mandatory for high-level evidence needed for incorporation into guidelines.

### Therapy

Recent retrospective data with ^177^Lu- / α-emitting-PSMA ligands show favorable safety and high efficiency exceeding those of other third-line systemic therapies in CRPC patients. Preliminary data suggest a significant survival benefit for patients treated with ^177^Lu-PSMA-617 or ^177^Lu-PSMA-I&T of several months. The theranostic approach urgently warrants future prospective studies.

## References

[1] ProstaScint. https://www.drugs.com/pro/prostascint.html. Accessed 13 June 2017.

[2] Bander NH, Trabulsi EJ, Kostakoglu L, Yao D, Vallabhajosula S, Smith-Jones P (2003). Targeting metastatic prostate cancer with radiolabeled monoclonal antibody J591 to the extracellular domain of prostate-specific membrane antigen. J Urol.

[3] Eder M, Schäfer M, Bauder-Wüst U, Hull WE, Wängler C, Mier W (2012). ^68^Ga-complex lipophilicity and the targeting property of a urea-based PSMA inhibitor for PET imaging. Bioconjug Chem.

[4] Afshar-Oromieh A, Haberkorn U, Eder M, Eisenhut M, Zechmann CM (2012). [^68^Ga]Gallium-labelled PSMA ligand as superior PET tracer for the diagnosis of PC: comparison with ^18^F–FECH. Eur J Nucl Med Mol Imaging.

[5] Pinto JT, Suffoletto BP, Berzin TM, Qiao CH, Lin S, Tong WP (1996). Prostate-specific membrane antigen: a novel folate hydrolase in human prostatic carcinoma cells. Clin Cancer Res.

[6] Israeli RS, Powell CT, Fair WR, Heston WD (1993). Molecular cloning of a complementary DNA encoding a prostate-specific membrane antigen. Cancer Res.

[7] Perner S, Hofer MD, Kim R, Shah RB, Li H, Möller P (2007). Prostate-specific membrane antigen expression as a predictor of prostate cancer progression. Hum Pathol.

[8] Ross JS, Sheehan CE, Fisher HA, Kaufman RP, Kaur P, Gray K (2003). Correlation of primary tumour prostate-specific membrane antigen expression with disease recurrence in prostate cancer. Clin Cancer Res.

[9] Gorin MA, Rowe SP, Hooper JE, Kates M, Hammers HJ, Szabo Z (2017). PSMA-targeted ^18^F-DCFPyL PET/CT imaging of clear cell renal cell carcinoma: results from a rapid autopsy. Eur Urol.

[10] Fendler WP, Eiber M, Beheshti M, Bomanji J, Ceci F, Cho S (2017). ^68^Ga-PSMA PET/CT: Joint EANM and SNMMI procedure guideline for PC imaging: version 1.0. Eur J Nucl Med Mol Imaging.

[11] Costello F, Langhorst S, Metter D, Palestro C, Zanzonico P. ACMUI Sub-Committee Report on Ge-68/Ga-68 Generator Licensing Guidance, Final Report, 8/25/16. https://www.nrc.gov/docs/ML1623/ML16238A311.pdf. Accessed 13 June 2017.

[12] National Cancer Institute, Surveillance, Epidemiology, and End Results Program. Cancer Stat Facts: Prostate Cancer. https://seer.cancer.gov/statfacts/html/prost.html. 21. July 2017.

[13] Mohler JL, Armstrong AJ, Bahnson RR, D’Amico AV, Davis BJ, Eastham JA (2016). Prostate cancer, version 1.2016. J Natl Compr Cancer Netw.

[14] Briganti A, Larcher A, Abdollah F, Capitanio U, Gallina A, Suardi N (2012). Updated nomogram predicting lymph node invasion in patients with prostate cancer undergoing extended pelvic lymph node dissection: the essential importance of percentage of positive cores. Eur Urol.

[15] Mottet N, Bellmunt J, Bolla M, Briers E, Cumberbatch MG, De Santis M (2017). EAU-ESTRO-SIOG guidelines on PC. Part 1: screening, diagnosis, and local treatment with curative intent. Eur Urol.

[16] Punnen S, Cooperberg MR, D’Amico AV, Karakiewicz PI, Moul JW, Scher HI (2013). Management of biochemical recurrence after primary treatment of prostate cancer: a systematic review of the literature. Eur Urol.

[17] Ceci F, Castellucci P, Mapelli P, Incerti E, Picchio M, Fanti S (2016). Evaluation of PC with ^11^C-choline PET/CT for treatment planning, response assessment, and prognosis. J Nucl Med.

[18] Parker C, Nilsson S, Heinrich D, Helle SI, O’Sullivan JM, Fosså SD (2013). Alpha emitter ^223^Ra and survival in metastatic prostate cancer. N Engl J Med.

[19] Pfannkuchen N, Meckel M, Bergmann R, Bachmann M, Bal C, Sathekge M, et al. Novel radiolabeled bisphosphonates for PET diagnosis and endoradiotherapy of bone metastases. Pharmaceuticals (Basel). 2017 May 18;10(2). 10.3390/ph10020045 PMC5490402 28524118

[20] Iagaru AH, Mittra E, Colletti PM, Jadvar H (2016). Bone-targeted imaging and radionuclide therapy in PC. J Nucl Med.

[21] Herrmann K, Larson SM, Weber WA (2017). Theranostic concepts: more than just a fashion trend—introduction and overview. J Nucl Med.

[22] Afshar-Oromieh A, Malcher A, Eder M, Eisenhut M, Linhart HG, Hadaschik BA (2013). PET imaging with a [^68^Ga]gallium-labelled PSMA ligand for the diagnosis of prostate cancer: biodistribution in humans and first evaluation of tumour lesions. Eur J Nucl Med Mol Imaging.

[23] Benešová M, Schäfer M, Bauder-Wüst U, Afshar-Oromieh A, Kratochwil C, Mier W (2015). Preclinical evaluation of a tailor-made DOTA-conjugated PSMA inhibitor with optimized linker moiety for imaging and endoradiotherapy of PC. J Nucl Med.

[24] Weineisen M, Schottelius M, Simecek J, Baum RP, Yildiz A, Beykan S (2015). ^68^Ga- and ^177^Lu-Labeled PSMA I&T: optimization of a PSMA-targeted theranostic concept and first proof-of-concept human studies. J Nucl Med.

[25] Robu S, Schottelius M, Eiber M, Maurer T, Gschwend J, Schwaiger M (2017). Preclinical evaluation and first patient application of ^99m^Tc-PSMA-I&S for SPECT imaging and radioguided surgery in PC. J Nucl Med.

[26] Hillier SM, Maresca KP, Lu G, Merkin RD, Marquis JC, Zimmerman CN (2013). ^99m^Tc-labeled small-molecule inhibitors of prostate-specific membrane antigen for molecular imaging of PC. J Nucl Med.

[27] Barrett JA, Coleman RE, Goldsmith SJ, Vallabhajosula S, Petry NA, Cho S (2013). First-in-man evaluation of 2 high-affinity PSMA-avid small molecules for imaging PC. J Nucl Med.

[28] Cho SY, Gage KL, Mease RC, Senthamizhchelvan S, Holt DP, Jeffrey-Kwanisai A (2012). Biodistribution, tumour detection, and radiation dosimetry of ^18^F-DCFBC, a low-molecular-weight inhibitor of prostate-specific membrane antigen, in patients with mPC. J Nucl Med.

[29] Chen Y, Pullambhatla M, Foss CA, Byun Y, Nimmagadda S, Senthamizhchelvan S (2011). 2-(3-{1-Carboxy-5-[(6-[18F]fluoro-pyridine-3-carbonyl)-amino]-pentyl}-ureido)-pentanedioic acid, [18F]DCFPyL, a PSMA-based PET imaging agent for PC. Clin Cancer Res.

[30] Cardinale J, Schäfer M, Benešová M, Bauder-Wüst U, Leotta K, Eder M (2017). Preclinical evaluation of ^18^F-PSMA-1007, a new prostate-specific membrane antigen ligand for prostate cancer imaging. J Nucl Med.

[31] Giesel FL, Hadaschik B, Cardinale J, Radtke J, Vinsensia M, Lehnert W (2017). ^18^F labelled PSMA-1007: biodistribution, radiation dosimetry and histopathological validation of tumour lesions in PC patients. Eur J Nucl Med Mol Imaging.

[32] Manyak MJ (2008). ^111^Indium capromab pendetide in the management of recurrent prostate cancer. Expert Rev Anticancer Ther.

[33] Pandit-Taskar N, O’Donoghue JA, Ruan S, Lyashchenko SK, Carrasquillo JA, Heller G (2016). First-in-human imaging with ^89^Zr-Df-IAB2M anti-PSMA minibody in patients with mPC: pharmacokinetics, biodistribution, dosimetry, and lesion-uptake. J Nucl Med.

[34] Kozikowski AP, Nan F, Conti P, Zhang J, Ramadan E, Bzdega T (2001). Design of remarkably simple, yet potent urea-based inhibitors of glutamate carboxypeptidase II (NAALADase). J Med Chem.

[35] Afshar-Oromieh A, Hetzheim H, Kratochwil C, Benesova M, Eder M, Neels OC (2015). The theranostic PSMA ligand PSMA-617 in the diagnosis of PC by PET/CT: biodistribution in humans, radiation dosimetry, and first evaluation of tumour lesions. J Nucl Med.

[36] Kratochwil C, Bruchertseifer F, Giesel FL, Weis M, Verburg FA, Mottaghy F, et al. ^225^Ac-PSMA-617 for PSMA-targeted α-radiation therapy of metastatic castration-resistant PC. J Nucl Med. 2016;57:1941–4. 10.2967/jnumed.116.178673 27390158

[37] Grubmüller B, Baum RP, Capasso E, Singh A, Ahmadi Y, Knoll P, et al. ^64^Cu-PSMA-617 PET/CT imaging of prostate adenocarcinoma: first in-human studies. Cancer Biother Radiopharm 2016 7. [Epub ahead of print]. 10.1089/cbr.2015.1964 27715146

[38] An open-label, single-arm, rater-blinded, multicenter phase ½ study to assess safety and diagnostic accuracy and radiotherapeutic implications of pre-operative Ga-68-PSMA-11 PET/CT imaging in comparison to histopathology, in newly diagnosed prostate cancer (PCA) patients at high risk for metastasis, scheduled for radical prostatectomy (RP) with extended pelvic lymph node dissection (EPLND) EudraCT No.: 2016001815–19.

[39] Sachpekidis C, Kopka K, Eder M, Hadaschik BA, Freitag MT, Pan L (2016). ^68^Ga-PSMA-11 dynamic PET/CT imaging in primary PC. Clin Nucl Med.

[40] Uprimny C, Kroiss AS, Decristoforo C, Fritz J, von Guggenberg E, Kendler D (2017). ^68^Ga-PSMA-11 PET/CT in primary staging of PC: PSA and GS predict the intensity of tracer accumulation in the primary tumour. Eur J Nucl Med Mol Imaging.

[41] Maurer T, Gschwend JE, Rauscher I, Souvatzoglou M, Haller B, Weirich G (2016). Diagnostic efficacy of ^68^Ga-PSMA PET compared to conventional imaging for LN-staging of 130 consecutive patients with intermediate to high-risk PC. J Urol.

[42] Budäus L, Leyh-Bannurah SR, Salomon G, Michl U, Heinzer H, Huland H (2016). Initial experience of ^68^Ga-PSMA PET/CT imaging in high-risk PC-patients prior to RP. Eur Urol.

[43] Fanti S, Minozzi S, Morigi JJ, Giesel F, Ceci F, Uprimny C, et al. Development of standardized image interpretation for ^68^Ga-PSMA PET/CT to detect prostate cancer recurrent lesions. Eur J Nucl Med Mol Imaging 2017 23. 10.1007/s00259-017-3725-1 28536833

[44] van Leeuwen PJ, Emmett L, Ho B, Delprado W, Ting F, Nguyen Q (2017). Prospective evaluation of ^68^Gallium-prostate-specific membrane antigen positron emission tomography/computed tomography for preoperative LN-staging in PC. BJU Int.

[45] Herlemann A, Wenter V, Kretschmer A, Thierfelder KM, Bartenstein P, Faber C (2016). ^68^Ga-PSMA positron emission tomography/computer tomography provides accurate staging of LN-regions prior to LN-dissection in patients with PC. Eur Urol.

[46] Demirkol MO, Acar Ö, Uçar B, Ramazanoğlu SR, Sağlıcan Y, Esen T (2015). Prostate-specific membrane antigen-based imaging in PC: impact on clinical decision making process. Prostate.

[47] Sterzing F, Kratochwil C, Fiedler H, Katayama S, Habl G, Kopka K (2016). ^68^Ga-PSMA-11 PET/CT: a new technique with high potential for the radiotherapeutic management of PC patients. Eur J Nucl Med Mol Imaging.

[48] Sahlmann CO, Meller B, Bouter C, Ritter CO, Ströbel P, Lotz J (2016). Biphasic ^68^Ga-PSMA-HBED-CC-PET/CT in patients with recurrent and high-risk PC. Eur J Nucl Med Mol Imaging.

[49] Perera M, Papa N, Christidis D, Wetherell D, Hofman MS, Murphy DG (2016). Sensitivity, specificity, and predictors of positive ^68^Ga-PSMA-PET in advanced PC: a systematic review and meta-analysis. Eur Urol.

[50] Ceci F, Uprimny C, Nilica B, Geraldo L, Kendler D, Kroiss A (2015). ^68^Ga-PSMA PET/CT for restaging recurrent prostate cancer: which factors are associated with PET/CT detection rate?. Eur J Nucl Med Mol Imaging.

[51] Verburg FA, Pfister D, Heidenreich A, Vogg A, Drude NI, Vöö S (2016). Extent of disease in recurrent prostate cancer determined by [^68^Ga]PSMA-HBED-CC PET/CT in relation to PSA levels, PSA doubling time and GS. Eur J Nucl Med Mol Imaging.

[52] Schiavina R, Ceci F, Romagnoli D, Uprimny C, Brunocilla E, Borghesi M (2015). ^68^Ga-PSMA-PET/CT-guided salvage retroperitoneal LN-dissection for disease relapse after radical prostatectomy for PC. Clin Genitourin Cancer.

[53] van Leeuwen PJ, Stricker P, Hruby G, Kneebone A, Ting F, Thompson B (2016). ^68^Ga-PSMA has a high detection rate of prostate cancer recurrence outside the prostatic fossa in patients being considered for salvage radiation treatment. BJU Int.

[54] Afshar-Oromieh A, Avtzi E, Giesel FL, Holland-Letz T, Linhart HG, Eder M (2015). The diagnostic value of PET/CT imaging with the ^68^Ga-labelled PSMA ligand HBED-CC in the diagnosis of recurrent prostate cancer. Eur J Nucl Med Mol Imaging.

[55] Afshar-Oromieh A, Holland-Letz T, Giesel FL, Kratochwil C, Mier W, Haufe S (2017). Diagnostic performance of ^68^Ga-PSMA-11 (HBED-CC) PET/CT in patients with recurrent prostate cancer: evaluation in 1007 patients. Eur J Nucl Med Mol Imaging.

[56] Eiber M, Maurer T, Souvatzoglou M, Beer AJ, Ruffani A, Haller B (2015). Evaluation of hybrid ^68^Ga-PSMA ligand PET/CT in 248 patients with BR after RP. J Nucl Med.

[57] Einspieler I, Ruascher I, Düwel C, Krönke M, Rischpler C, Habl G (2017). Detection efficacy of hybrid 68Ga-PSMA ligand PET/CT in PC-patients with biochemical recurrence after primary radiation therapy defined by phoenix criteria. J Nucl Med.

[58] Kabasakal L, Demirci E, Nematyazar J, Akyel R, Razavi B, Ocak M (2017). The role of PSMA PET/CT imaging in restaging PC patients with low prostate-specific antigen levels. Nucl Med Commun.

[59] Sachpekidis C, Eder M, Kopka K, Mier W, Hadaschik BA, Haberkorn U (2016). ^68^Ga-PSMA-11 dynamic PET/CT imaging in biochemical relapse of PC. Eur J Nucl Med Mol Imaging.

[60] Hövels AM, Heesakkers RA, Adang EM, Jager GJ, Jager GJ, Strum S, Hoogeveen YL (2008). The diagnostic accuracy of CT and MRI in the staging of pelvic LN in patients with PC: a meta-analysis. Clin Radiol.

[61] Rauscher I, Maurer T, Beer AJ, Graner FP, Haller B, Weirich G (2016). Value of ^68^Ga-PSMA HBED-CC for the assessment of LN metastases in PC-patients with BR: comparison with histopathology after salvage lymphadenectomy. J Nucl Med.

[62] Albisinni S, Artigas C, Aoun F, Biaou I, Grosman J, Gil T (2017). Clinical impact of ^68^Ga-prostate-specific membrane antigen (PSMA) positron emission tomography/computed tomography (PET/CT) in patients with prostate cancer with rising prostate-specific antigen after treatment with curative intent: preliminary analysis of a multidisciplinary approach. BJU Int.

[63] Lütje S, Blex S, Gomez B, Schaarschmidt BM, Umutlu L, Forsting M (2016). Optimization of acquisition time of ^68^Ga-PSMA-ligand PET/MRI in patients with local and mPC. PLoS One.

[64] Noto B, Büther F, Auf der Springe K, Avramovic N, Heindel W, Schäfers M, et al. Impact of PET acquisition durations on image quality and lesion detectability in whole-body ^68^Ga-PSMA PET/MRI. EJNMMI Res. 2017;7:–12. 10.1186/s13550-017-0261-8 PMC5293699 28168589

[65] Afshar-Oromieh A, Wolf M, Haberkorn U, Kachelrieß M, Gnirs R, Kopka K, et al. Effects of arm truncation on the appearance of the halo artifact in ^68^Ga-PSMA-11 (HBED-CC) PET/MRI. Eur J Nucl Med Mol Imaging 2017 15. 10.1007/s00259-017-3718-0 28508120

[66] Eiber M, Weirich G, Holzapfel K, Souvatzoglou M, Haller B, Rauscher I (2016). Simultaneous ^68^Ga-PSMA HBED-CC PET/mpMRI improves the localization of primary PC. Eur Urol.

[67] Maurer T, Gschwend J, Wester HJ, Souvatzoglou M, Beer A, Holzapfel K. PET imaging with of prostate-specific membrane antigen (PSMA) for staging of primary prostate cancer with ^68^Ga-HBED-PSMA. J Clin Oncol. 10.1200/jco.2015.33.15_suppl.e16038.

[68] Giesel FL, Sterzing F, Schlemmer HP, Holland-Letz T, Mier W, Rius M (2016). Intra-individual comparison of ^68^Ga-PSMA-11-PET/CT and multi-parametric MR for imaging of primary prostate cancer. Eur J Nucl Med Mol Imaging.

[69] Zamboglou C, Drendel V, Jilg CA, Rischke HC, Beck TI, Schultze-Seemann W (2017). Comparison of ^68^Ga-HBED-CC PSMA-PET/CT and multiparametric MRI for gross tumour volume detection in patients with primary PC based on slice by slice comparison with histopathology. Theranostics.

[70] Zamboglou C, Wieser G, Hennies S, Rempel I, Kirste S, Soschynski M (2016). MRI versus ^68^Ga-PSMA PET/CT for gross tumour volume delineation in radiation treatment planning of primary prostate cancer. Eur J Nucl Med Mol Imaging.

[71] Iagaru A (2016). ^68^Ga-PSMA PET/MRI for detection of regional nodal and distant metastases in patients with intermediate and high-risk PC. [PROS0075; NCT02678351]. Anco Fax News.

[72] Kranzbühler B, Nagel H, Becker AS, Müller J, Huellner M, Stolzmann P, et al. Clinical performance of ^68^Ga-PSMA-11 PET/MRI for the detection of recurrent prostate cancer following radical prostatectomy. Eur J Nucl Med Mol Imaging 2018;45(1):20–30. 10.1007/s00259-017-3850-x 29032394

[73] Freitag MT, Radtke JP, Hadaschik BA, Kopp-Schneider A, Eder M, Kopka K (2016). Comparison of hybrid ^68^Ga-PSMA PET/MRI and ^68^Ga-PSMA PET/CT in the evaluation of LN- and bone metastases of PC. Eur J Nucl Med Mol Imaging.

[74] Freitag MT, Radtke JP, Afshar-Oromieh A, Roethke MC, Hadaschik BA, Gleave M (2017). Local recurrence of PC after RP is at risk to be missed in ^68^Ga-PSMA-11-PET of PET/CT and PET/MRI: comparison with mpMRI integrated in simultaneous PET/MRI. Eur J Nucl Med Mol Imaging.

[75] Maurer T, Beck V, Beer A, Souvatzoglou M, Holzapfel K, Kübler H (2015). Detection rates of ^68^Ga-labelled ligand of PSMA PET/CT and PET/MRI in 332 consecutive patients with biochemical recurrency after RP. J Urol.

[76] Castellucci P, Fuccio C, Nanni C, Santi I, Rizzello A, Lodi F, Franceschelli A, Martorana G, Manferrari F, Fanti S (2009). Influence of trigger PSA and PSA kinetics on ^11^C-Choline PET/CT detection rate in patients with biochemical relapse after radical prostatectomy. J Nucl Med.

[77] Graziani T, Ceci F, Castellucci P, Polverari G, Lima GM, Lodi F (2016). ^11^C-Choline PET/CT for restaging prostate cancer. Results from 4,426 scans in a single-centre patient series. Eur J Nucl Med Mol Imaging.

[78] Fanti S, Minozzi S, Castellucci P, Balduzzi S, Herrmann K, Krause BJ (2016). PET/CT with ^11^C-choline for evaluation of prostate cancer patients with biochemical recurrence: meta-analysis and critical review of available data. Eur J Nucl Med Mol Imaging.

[79] Evangelista L, Guttilla A, Zattoni F, Zattoni F (2013). Utility of choline positron emission tomography/computed tomography for lymph node involvement identification in intermediate- to high-risk PC: a systematic literature review and meta-analysis. Eur Urol.

[80] Pfister D, Porres D, Heidenreich A, Heidegger I, Knuechel R, Steib F (2016). Detection of recurrent PC-lesions before salvage lymphadenectomy is more accurate with ^68^Ga-PSMA-HBED-CC than with ^18^F-fluoroethylcholine PET/CT. Eur J Nucl Med Mol Imaging.

[81] Afshar-Oromieh A, Zechmann CM, Malcher A, Eder M, Eisenhut M, Linhart HG (2014). Comparison of PET imaging with a ^68^Ga-labelled PSMA ligand and ^18^F-choline-based PET/CT for the diagnosis of recurrent prostate cancer. Eur J Nucl Med Mol Imaging.

[82] Bluemel C, Krebs M, Polat B, Linke F, Eiber M, Samnick S (2016). ^68^Ga-PSMA-PET/CT in patients with biochemical prostate cancer recurrence and negative ^18^F-choline-PET/CT. Clin Nucl Med.

[83] von Eyben FE, Picchio M, von Eyben R, Rhee H, Bauman G. ^68^Ga-labeled prostate-specific membrane antigen ligand positron emission tomography/computed tomography for PC: a systematic review and meta-analysis. Eur Urol Focus. 2016. 10.1016/j.euf.2016.11.002 28753806

[84] Giovacchini G, Giovannini E, Leoncini R, Riondato M, Ciarmiello A. PET and PET/CT with radiolabeled choline in prostate cancer: a critical reappraisal of 20 years of clinical studies. Eur J Nucl Med Mol Imaging. 2017; 44(10):1751–76. 10.1007/s00259-017-3700-x 28409220

[85] Morigi JJ, Stricker PD, van Leeuwen PJ, Tang R, Ho B, Nguyen Q (2015). Prospective comparison of ^18^F-fluoromethylcholine versus ^68^Ga-PSMA PET/CT in PC-patients who have rising PSA after curative treatment and are being considered for targeted therapy. J Nucl Med.

[86] Afshar-Oromieh A, Sattler LP, Mier W, Hadaschik BA, Debus J, Holland-Letz T (2017). The clinical impact of additional late PET/CT imaging with ^68^Ga-PSMA-11 (HBED-CC) in the diagnosis of PC. J Nucl Med.

[87] Schmuck S, Nordlohne S, von Klot CA, Henkenberens C, Sohns JM, Christiansen H (2017). Comparison of standard and delayed imaging to improve the detection rate of [^68^Ga]PSMA I&T PET/CT in patients with biochemical recurrence or prostate-specific antigen persistence after primary therapy for PC. Eur J Nucl Med Mol Imaging.

[88] Uprimny C, Kroiss AS, Decristoforo C, Fritz J, Warwitz B, Scarpa L (2017). Early dynamic imaging in ^68^Ga- PSMA-11 PET/CT allows discrimination of urinary bladder activity and PC-lesions. Eur J Nucl Med Mol Imaging.

[89] Kabasakal L, Demirci E, Ocak M, Akyel R, Nematyazar J, Aygun A (2015). Evaluation of PSMA PET/CT imaging using a ^68^Ga-HBED-CC ligand in patients with PC and the value of early pelvic imaging. Nucl Med Commun.

[90] Fendler WP, Calais J, Allen-Auerbach M, Bluemel C, Eberhardt N, Emmett L, et al. ^68^Ga-PSMA-11 PET/CT interobserver agreement for PC-assessments: an international multicenter prospective study. J Nucl Med 2017; 58(10):1617–23. 10.2967/jnumed.117.190827 28408531

[91] Wright GL, Grob BM, Haley C, Grossman K, Newhall K, Petrylak D (1996). Upregulation of prostate-specific membrane antigen after androgen-deprivation therapy. Urology.

[92] Hope TA, Truillet C, Ehman EC, Afshar-Oromieh A, Aggarwal R, Ryan CJ (2017). ^68^Ga-PSMA-11 PET imaging of response to androgen receptor inhibition: first human experience. J Nucl Med.

[93] Silver DA, Pellicer I, Fair WR, Heston WD, Cordon-Cardo C (1997). Prostate-specific membrane antigen expression in normal and malignant human tissues. Clin Cancer Res.

[94] Chang SS, O’Keefe DS, Bacich DJ, Reuter VE, Heston WD, Gaudin PB (1999). Prostate-specific membrane antigen is produced in tumour-associated neovasculature. Clin Cancer Res.

[95] Krohn T, Verburg FA, Pufe T, Neuhuber W, Vogg A, Heinzel A (2015). [^68^Ga]PSMA-HBED uptake mimicking LN-metastasis in coeliac ganglia: an important pitfall in clinical practice. Eur J Nucl Med Mol Imaging.

[96] Beauregard JM, Hofman MS, Kong G, Hicks RJ (2012). The tumour sink effect on the biodistribution of ^68^Ga-DOTA-octreotate: implications for peptide receptor radionuclide therapy. Eur J Nucl Med Mol Imaging.

[97] Pandey MK, Byrne JF, Jiang H, Packard AB, DeGrado TR (2014). Cyclotron production of ^68^Ga via the ^68^Zn(p,n)^68^Ga reaction in aqueous solution. Am J Nucl Med Mol Imaging.

[98] Kelly J, Amor-Coarasa A, Nikolopoulou A, Kim D, Williams C, Ponnala S (2017). Synthesis and pre-clinical evaluation of a new class of high-affinity ^18^F-labeled PSMA ligands for detection of PC by PET imaging. Eur J Nucl Med Mol Imaging.

[99] Li X, Rowe SP, Leal JP, Gorin MA, Allaf ME, Ross AE (2017). Semiquantitative parameters in PSMA-targeted PET-imaging with ^18^F-DCFPyL: variability in normal-organ uptake. J Nucl Med.

[100] Dietlein F, Kobe C, Neubauer S, Schmidt M, Stockter S, Fischer T (2017). PSA-stratified performance of ^18^F- and ^68^Ga-PSMA PET in patients with biochemical recurrence of prostate cancer. J Nucl Med.

[101] Kesch C, Vinsensia M, Radtke JP, Schlemmer HP, Heller M, Ellert E, et al. Intra-individual comparison of ^18^F-PSMA-1007-PET/CT, multi-parametric MRI and radical prostatectomy specimen in patients with primary PC—a retrospective, proof of concept study. J Nucl Med. 2017;58(11):1805–10. 10.2967/jnumed.116.189233 28473595

[102] Giesel FL, Cardinale J, Schäfer M, Neels O, Benešová M, Mier W (2016). ^18^F-labelled PSMA-1007 shows similarity in structure, biodistribution and tumour uptake to the theragnostic compound PSMA-617. Eur J Nucl Med Mol Imaging.

[103] Gorin MA, Rowe SP, Mana-ay M (2016). Study of PSMA-targeted ^18^F-DCFPyL PET/CT in the evaluation of men with an elevated PSA following RP. J Urol.

[104] Cantiello F, Gangemi V, Cascini GL, Calabria F, Moschini M, Ferro M, et al. Diagnostic accuracy of ^64^copper prostate-specific membrane antigen positron emission tomography/computed tomography for primary LN-staging of intermediate- to high-risk PC: our preliminary experience. Urology 2017;105:139–145. 10.1016/j.urology.2017.04.019 28438628

[105] Krause BJ, Souvatzoglou M, Tuncel M, Herrmann K, Buck AK, Praus C (2008). The detection rate of [^11^C]choline-PET/CT depends on the serum PSA-value in patients with BR of PC. Eur J Nucl Med Mol Imaging.

[106] Picchio M, Spinapolice EG, Fallanca F, Crivellaro C, Giovacchini G, Gianolli L (2012). [^11^C]Choline PET/CT detection of bone metastases in patients with PSA progression after primary treatment for prostate cancer: comparison with bone scintigraphy. Eur J Nucl Med Mol Imaging.

[107] Mamede M, Ceci F, Castellucci P, Schiavina R, Fuccio C, Nanni C (2013). The role of ^11^C-choline PET imaging in the early detection of recurrence in surgically treated prostate cancer patients with very low PSA level <0.5 ng/mL. Clin Nucl Med.

[108] Passoni NM, Suardi N, Abdollah F, Picchio M, Giovacchini G, Messa C (2014). Utility of [^11^C]choline PET/CT in guiding lesion-targeted salvage therapies in patients with prostate cancer recurrence localized to a single lymph node at imaging: results from a pathologically validated series. Urol Oncol.

[109] Mitchell CR, Lowe VJ, Rangel LJ, Hung JC, Kwon ED, Karnes RJ (2013). Operational characteristics of ^11^C-choline positron emission tomography/computerized tomography for prostate cancer with biochemical recurrence after initial treatment. J Urol.

[110] Cornford P, Bellmunt J, Bolla M, Briers E, De Santis M, Gross T (2017). EAU-ESTRO-SIOG guidelines on PC. Part II: treatment of relapsing, metastatic, and CRPC. Eur Urol.

[111] Baum RP, Kulkarni HR, Schuchardt C, Singh A, Wirtz M, Wiessalla S (2016). Lutetium-177 PSMA radioligand therapy of mCRPC: safety and efficacy. J Nucl Med.

[112] Kulkarni HR, Singh A, Schuchardt C, Niepsch K, Sayeg M, Leshch Y (2016). PSMA-based radioligand therapy for mCRPC: the Bad Berka experience since 2013. J Nucl Med.

[113] Rahbar K, Ahmadzadehfar H, Kratochwil C, Haberkorn U, Schäfers M, Essler M (2017). German multicenter study investigating ^177^Lu-PSMA-617 radioligand therapy in advanced PC-patients. J Nucl Med.

[114] Rahbar K, Schmidt M, Heinzel A, Eppard E, Bode A, Yordanova A (2016). Response and tolerability of a single activity of ^177^Lu-PSMA-617 in patients with mCRPC: a multicenter retrospective analysis. J Nucl Med.

[115] Yadav MP, Ballal S, Tripathi M, Damle NA, Sahoo RK, Seth A (2017). ^177^Lu-DKFZ-PSMA-617 therapy in mCRPC: safety, efficacy, and quality of life assessment. Eur J Nucl Med Mol Imaging.

[116] Heck MM, Retz M, D’Alessandria C, Rauscher I, Scheidhauer K, Maurer T (2016). Systemic radioligand therapy with ^177^Lu-labeled prostate-specific membrane antigen-ligand for imaging and therapy in patients with mCRPC. J Urol.

[117] Fendler WP, Reinhardt S, Ilhan H, Delker A, Böning G, Gildehaus FJ (2017). Preliminary experience with dosimetry, response and patient reported outcome after ^177^Lu-PSMA-617 therapy for mCRPC. Oncotarget.

[118] Scarpa L, Buxbaum S, Kendler D, Fink K, Bektic J, Gruber L (2017). The ^68^Ga/^177^Lu theragnostic concept in PSMA targeting of CRPC: correlation of SUV_max_ values and absorbed activity estimates. Eur J Nucl Med Mol Imaging.

[119] Kratochwil C, Giesel FL, Stefanova M, Benešová M, Bronzel M, Afshar-Oromieh A (2016). PSMA-targeted radionuclide therapy of mCRPC with ^177^Lu-labeled PSMA-617. J Nucl Med.

[120] Ahmadzadehfar H, Eppard E, Kürpig S, Fimmers R, Yordanova A, Schlenkhoff CD (2016). Therapeutic response and side effects of repeated radioligand therapy with ^177^Lu-PSMA-DKFZ-617 of mCRPC. Oncotarget.

[121] Ahmadzadehfar H, Wegen S, Yordanova A, Fimmers R, Kürpig S, Eppard E (2017). Overall survival and response pattern of mCRPC to multiple cycles of radioligand therapy using ^177^Lu-PSMA-617. Eur J Nucl Med Mol Imaging.

[122] Yordanova A, Becker A, Eppard E, Kürpig S, Fisang C, Feldmann G (2017). The impact of repeated cycles of radioligand therapy using ^177^Lu-PSMA-617 on renal function in patients with hormone refractory mPC. Eur J Nucl Med Mol Imaging.

[123] Bräuer A, Grubert LS, Roll W, Schrader AJ, Schäfers M, Bögemann M, et al. ^177^Lu-PSMA-617 radioligand therapy and outcome in patients with metastasised castration-resistant prostate cancer. Eur J Nucl Med Mol Imaging. 2017; 10.1007/s00259-017-3751-z. 10.1007/s00259-017-3751-z 28624848

[124] Ahmadzadehfar H, Zimbelmann S, Yordanova A, Fimmers R, Kürpig S, Eppard E, et al. Radioligand therapy of metastatic prostate cancer using ^177^Lu-PSMA-617 after radiation exposure to ^223^Ra-dichloride. Oncotarget 2017. [Epub ahead of print]. 10.18632/oncotarget.15698 PMC5589682 28903443

[125] Tesson M, Rae C, Nixon C, Babich JW, Mairs RJ (2016). Preliminary evaluation of prostate-targeted radiotherapy using ^131^I-MIP-1095 in combination with radiosensitising chemotherapeutic drugs. J Pharm Pharmacol.

[126] Afshar-Oromieh A, Haberkorn U, Zechmann C, Armor T, Mier W, Spohn F (2017). Repeated PSMA-targeting radioligand therapy of metastatic prostate cancer with ^131^I-MIP-1095. Eur J Nucl Med Mol Imaging.

[127] Kiess AP, Minn I, Chen Y, Hobbs R, Sgouros G, Mease RC (2015). Auger radiopharmaceutical therapy targeting prostate-specific membrane antigen. J Nucl Med.

[128] Kiess A, Minn IL, Vaidyanathan G, Hobbs RF, Josefsson A, Shen C (2016). (2S)-2-(3-(1-Carboxy-5-(4-211At-astatobenzamido)pentyl)ureido)-pentanedioic acid for PSMA-targeted α-particle radiopharmaceutical therapy. J Nucl Med.

[129] Kratochwil C, Bruchertseifer F, Rathke H, Bronzel M, Apostolidis C, Weichert W (2017). Targeted alpha therapy of mCRPC with ^225^Actinium-PSMA-617: dosimetry estimate and empirical activity finding. J Nucl Med.

[130] Sathekge M, Knoesen O, Meckel M, Modiselle M, Vorster M, Marx S (2017). ^213^Bi-PSMA-617 targeted alpha-radionuclide therapy in mCRPC. Eur J Nucl Med Mol Imaging.

[131] World Medical Association (2013). World medical association Declaration of Helsinki: ethical principles for medical research involving human subjects. JAMA.

[132] Kabasakal L, AbuQbeitah M, Aygün A, Yeyin N, Ocak M, Demirci E (2015). Pre-therapeutic dosimetry of normal organs and tissues of ^177^Lu-PSMA-617 prostate-specific membrane antigen (PSMA) inhibitor in patients with CRPC. Eur J Nucl Med Mol Imaging.

[133] Kratochwil C, Giesel FL, Leotta K, Eder M, Hoppe-Tich T, Youssoufian H (2015). PMPA for nephroprotection in PSMA-targeted radionuclide therapy of PC. J Nucl Med.

[134] Okamoto S, Thieme A, Allmann J, D’Alessandria C, Maurer T, Retz M (2017). Radiation dosimetry for ^177^Lu-PSMA-I&T in mCRPC: absorbed activity in normal organs and tumour lesions. J Nucl Med.

[135] Bohn KP, Kletting P, Solbach C, Beer AJ, Krohn T, Effekt der Kühlung v (2017). Speicheldrüsen bei der Therapie mit PSMA-Radioliganden. Nuklearmedizin.

[136] Li Y, Taylor JMG, Ten Haken RK, Eisbruch A (2007). The impact of activity on parotid salivary recovery in head and neck cancer patients treated with radiation therapy. Int J Radiat Oncol Biol Phys.

[137] Emami B, Lyman J, Brown A, Coia L, Goitein M, Munzenrider JE (1991). Tolerance of normal tissue to therapeutic irradiation. Int J Radiat Oncol Biol Phys.

[138] Kratochwil C, Giesel FL, Eder M, Afshar-Oromieh A, Benešová M, Mier W (2015). ^177^Lu-labelled PSMA ligand-induced remission in a patient with mPC. Eur J Nucl Med Mol Imaging.

[139] Ahmadzadehfar H, Rahbar K, Kürpig S, Bögemann M, Claesener M, Eppard E (2015). Early side effects and first results of radioligand therapy with ^177^Lu-DKFZ-617 PSMA of mCRPC: a two-centre study. EJNMMI Res.

[140] von Eyben FE, Roviello G, Kiljunen T, Uprimny C, Virgolini I, Kairemo K, Joensuu T. Is third-line treatment of metastatic castration-resistant prostate cancer better than ^177^Lu-PSMA radioligand therapy? A systematic review and meta-analysis. Submitted to EJNMMI. 10.1007/s00259-017-3895-x PMC5787223 29247284

[141] Fendler WP, Stuparu AD, Evans-Axelsson S, Lückerath K, Wei L, Kim W, et al. Establishing ^177^Lu-PSMA-617 radioligand therapy in a syngeneic model of murine PC. JNM 2017. 10.2967/jnumed.117.193359 PMC6944167 28546332

[142] Parker CC, Coleman RE, Sartor O, Vogelzang NJ, Bottomley D, Heinrich D, et al. Three-year safety of ^223^Ra dichloride in patients with CRPC and symptomatic bone metastases from phase III randomized alpharadin in symptomatic prostate cancer trial. Eur Urol. 2017 Jul 10; 10.1016/j.eururo.2017.06.021. [Epub ahead of print] 10.1016/j.eururo.2017.06.021 28705540

[143] Florimonte L, Dellavedova L, Maffioli LS (2016). ^223^Radium dichloride in clinical practice: a review. Eur J Nucl Med Mol Imaging.

[144] ALGETA ASA: Alpharadion ® injection (radium-223 chloride) – Investigator’s brochure, Oslo, Norway Edition No: 7, 15 2010.

[145] Chittenden SJ, Hindorf C, Parker CC, Lewington VJ, Pratt BE, Johnson B (2015). A phase 1, open-label study of the biodistribution, pharmacokinetics, and dosimetry of ^223^Ra-dichloride in patients with hormone-refractory prostate cancer and skeletal metastases. J Nucl Med.

[146] Lassmann M, Nosseke D (2013). Dosimetry of ^223^Ra-chloride: use to normal organs and tissues. Eur J Nucl Med Mol Imaging.

[147] ICRP. Age-dependent activities to members of the public from intake of radionuclides: Part 2. Ingestion activity coefficients. A report of a task Group of Committee 2 of the international commission on radiological protection. Ann ICRP. 1993;23:1–167. 7978694

[148] Sgourus G, Roeske JC, McDevitt MR, Palm S, Allen BJ, Fisher DR (2010). MIRD pamphlet no.22 (abridged): radiobiology and dosimetry of alpha-particle emitters for targeted radionuclide therapy. J Nucl Med.

